# Survival, morbidity, and quality of life in pulmonary arterial hypertension patients: a systematic review of outcomes reported by population-based observational studies

**DOI:** 10.1186/s12931-024-02994-w

**Published:** 2024-10-16

**Authors:** Stefan Reinders, Eva-Maria Didden, Rose Ong

**Affiliations:** 1 Independent Consultant, Lima, Peru; 2 Actelion Pharmaceuticals Ltd, A Johnson & Johnson Company, Global Epidemiology, Allschwil, Switzerland

**Keywords:** Pulmonary arterial hypertension, Systematic review, Survival, Morbidity, Observational studies

## Abstract

**Background:**

Comprehensive summaries on real-world outcomes in pulmonary arterial hypertension (PAH)—a rare, incurable condition, are lacking.

**Main body of the abstract:**

We conducted a systematic literature review to describe current survival, morbidity, and quality of life (QoL) outcomes in adult and pediatric PAH patients. We searched Medline and Embase electronic databases, clinicaltrials.gov, and encepp.eu entries, and grey literature to identify outcome estimates for right-heart catheterization-confirmed PAH patients from population-based observational studies (search date: 25 Nov 2021). Data were synthesized using a narrative approach and post-hoc subgroup meta-analyses were conducted to explore adult survival by region, disease severity, representativeness, and study period. The search yielded 7473 records. Following screening and full text review, 22 unique studies with 31 individual reports of outcomes were included. Studies were mostly national registries (n = 21), European (n = 13) and covering adults (n = 17); only six had systematic countrywide coverage of centers. Survival was the most frequently reported outcome (n = 22). Global adult 1-, 3-, and 5-year survival ranged from 85 to 99% (n = 15), 65 to 95% (n = 14), and 50 to 86% (n = 9), respectively. Subgroup meta-analysis showed that 1-, 3-, and 5-year survival in Europe was 90% (95% CI 86–94%; n = 8), 78% (95% CI 68–86%; n = 8), and 61% (95% CI 49–72%; n = 6), respectively; 1-year survival in North America was 88% (95% CI 83–93%; n = 3) and 3-year survival in Asia was 85% (95% CI 82–88%; n = 3). No difference in survival between regions was observed. Subgroup analysis suggested higher survival in patients with better baseline functional class; however, interpretation should be cautioned due to large subgroup heterogeneity and potential missingness of data.

**Short conclusion:**

This review describes current disease outcomes based on well-defined and representative PAH populations. There is an overall lack of follow-up data for morbidity and QoL outcomes; survival estimates for pediatric patients are scarce and may not be generalizable to the current treatment era, although publications from large pediatric registries became available after our search date. This study demonstrated a remaining unmet need world-wide to improve long-term prognosis in PAH in the current era.

**Supplementary Information:**

The online version contains supplementary material available at 10.1186/s12931-024-02994-w.

## Introduction

Pulmonary hypertension (PH) is a chronic and progressive disease characterized by abnormally-high blood pressure in the lungs that can ultimately lead to right ventricular failure and death [1]. Pulmonary arterial hypertension (PAH) is a rare and incurable subgroup of PH, estimated to have an annual incidence of 5.8 adults per million, and a prevalence of 47.6 to 54.7 adults per million, based on mostly European data [2]. The median survival of untreated patients with PAH is 2.8 years [3]. Since the 3rd World Symposium of Pulmonary Hypertension (WSPH) held in 2003, PAH was hemodynamically defined by the presence of mean pulmonary arterial pressure (mPAP) > 25 mmHg with a pulmonary arterial wedge pressure (PAWP) ≤ 15 mmHg and elevated pulmonary vascular resistance (PVR) > 3 Wood Units (WU) [4]. The 6th WSPH in 2018 proposed a change to the mPAP threshold to > 20 mmHg, which is now reflected in the latest European Society of Cardiology and the European Respiratory Society (ESC/ERS) guideline [5], as well as an updated PVR threshold (> 2 WU), based on better characterization of the upper limits of normal.

Since the approval of the first targeted treatment for PAH, epoprostenol in 1995 [6], more than ten pharmacologic therapies have been licensed for the treatment of patients with PAH [7]. Macitentan, selexipag, riociguat, and treprostinil have all been approved by the Food and Drug Administration (FDA) in the last decade [8–15]. However, despite recent advances in the treatment landscape for PAH, there are often delays in the diagnosis and treatment of PAH, and the disease remains incurable [16]. The ESC/ERS guidelines recommend assessing patient risk of 1-year mortality by measuring a combination of prognostic predictors to guide treatment decisions [5, 17]. Several risk stratification models have been developed since the recommendation by the ESC/ERS, including those based on the Comparative, Prospective Registry of Newly Initiated Therapies for Pulmonary Hypertension (COMPERA) [18, 19], and the Registry to Evaluate Early and Long-term PAH disease management (REVEAL) [20]. These 3- or 4-strata models incorporate morbidity-related variables such as 6-min walk distance (6MWD), World Health Organization (WHO), functional class (FC), and biomarkers (brain natriuretic peptide) (BNP), and the N-terminal fragment of proBNP (NT-proBNP) as key components of their risk assessments.

Monitoring of disease outcomes is vital for the direction of future research. However, the rarity of PAH means that outcome data are often based on disparate and relatively small cohorts of patients [21]. Furthermore, although the ESC/ERS guidelines state that PAH must be diagnosed by right-heart catheterization (RHC) [5], in clinical practice, PAH diagnoses are not always confirmed by this method [22], which may introduce heterogeneity when comparing studies. Study findings may also vary due to the representativeness of the patient cohort. For example, the findings from a single-center study that is not representative of the national population may be different to that of a study involving all centers in a given country [2, 23].

A recent systematic literature review [24] estimated prevalence, incidence, and survival in PAH and reported a 1-year survival ranging from 67 to 99% across all studies. This wide range of survival is likely explained by the large heterogeneity of included studies, with differences regarding participants characteristics, diagnostic criteria of PH, disease severity, geographic region, study period (including coverage of prevalent and/or incident patients with different years of diagnosis), and study representativeness. The authors did not assess other outcomes besides survival, nor are we aware of any other systematic reviews that does. An understanding of morbidity outcomes and patients’ quality of life (QoL) is, however, crucial for a comprehensive understanding of current prognosis in PAH [25–29].

There is a need for a comprehensive overview of disease outcomes based on well-defined, comparable, and representative PAH populations. We conducted a systematic review to describe survival, morbidity, and QoL outcomes in adult and pediatric patients with RHC-confirmed and 3rd WSPH-classified PAH, as reported by population-based observational studies. We also conducted post-hoc subgroup meta-analyses to stratify and explore differences in survival by relevant subgroups including geographic region, disease severity, study representativeness, and study period.

Methods

### Search strategy

We conducted an electronic database search on 25 Nov 2021. We systematically searched articles and conference abstracts via OvidSP (Medline and Embase), using three search blocks: (1) population (patients with PH, including PAH); (2) outcomes (survival, morbidity, and/or QoL), and; (3) study design (observational studies, including registries, cohorts, databases, and chart reviews). We searched clinicaltrials.gov and encepp.eu for relevant observational studies using the keyword ‘pulmonary hypertension’. We also conducted a grey literature search via Google, searched websites of PH registries, PH patient organizations, bibliographies of seminal reviews of PH registries [2, 24, 30, 31] and studies included in this review (see Supplementary Method 1 for detailed methods).

### Selection of studies

We defined eligibility criteria in line with the aims of this review, ensuring that the outcome estimates were based on a well-defined, comparable, and representative PAH population; therefore, the search included population-based studies covering PAH patients diagnosed by RHC (exclusively or partially) and outcomes of interest (Table 1). Titles and abstracts of articles and other documents identified in the searches were then screened to identify potentially relevant studies, followed by a full text review to confirm final eligibility (see Supplementary Method 2 for detailed methods).

**Table 1:** Eligibility criteria for inclusion of studies into the review

Inclusion	(1) Population ▪ PH Group 1 confirmed by right-heart catheterization (RHC) using 3rd WSPH Venice classification [81] or later (including studies with exclusive or partial diagnoses by RHC and echocardiography) ▪ Any age, including adults and children (2) Outcomes ▪ Primary outcome: survival ▪ Secondary outcomes: • Morbidity: risk scores, hospitalization, FC, 6MWD, BNP/NT-proBNP, transplantation, oxygen use, prostacyclin analog use potentially associated with disease progression • QoL (3) Study type ▪ Any observational multi-center study or single-center study with national representativeness
Exclusion	(1) Principal disease under investigation not PAH, no RHC-confirmed PAH using 3rd WSPH classification system or later, or PH Groups 2–5 (2) Relevant outcomes not reported (3) Clinical trials, open-label interventional studies, and studies investigating the effect of a specific treatment or procedure (4) Type of communication: in vitro, case report, case series, editorial, letter, review, protocols, animal studies, guidelines (5) Selected subpopulations or individual PH subaetiology not representative of entire PH Group (6) Small studies with a total sample size below n = 30 (7) Duplicate references (8^a^) Single-center studies with regional representativeness or no defined catchment area (9^a^) Outcomes for the same or earlier time period already reported by other reference of the same study (10^a^) Study population already covered by a larger and/or more representative study (11^a^) No results yet reported and/or insufficient information for characterization

^a^Criteria only applied during full text review

*6MWD* 6-min walk distance, *BNP* brain natriuretic peptide, *FC* functional class, *NT*-*proBNP* N-terminal pro-brain natriuretic peptide, *PAH* pulmonary arterial hypertension, *PH* pulmonary hypertension, *QoL* quality of life, *RHC* right-heart catheterization, *WSPH* World Symposium of Pulmonary Hypertension

### Data collection

Data from eligible studies were collected using standardized forms covering general information, details on the study population, baseline characteristics (at diagnosis or enrollment, as reported by authors), and outcomes at follow-up. Survival estimates were collected as reported by authors or extracted from Kaplan–Meier curves using Engauge Digitizer 12.0 [32]. Estimates of morbidity or QoL were collected for baseline, follow-up or change from baseline to follow-up (see Supplementary Method 3 for detailed methods).

### Assessment of representativeness

We assessed representativeness of studies using an adapted classification system based on definitions developed by Leber et al. [2]. Studies that covered all national PH expert or referral centers were classified as ‘national, systematic’. Studies that had large geographic coverage of most regions nationally and/or were described by the authors as ‘national’, but with incomplete coverage of all centers on a national level, were classified as ‘national, non-systematic’. Other studies were classified as ‘non-national’ and studies covering multiple countries were classified ‘multi-national’ (see Supplementary Method 4 for detailed methods).

### Data reporting

We characterized all included studies with respect to characteristics that we considered most important to reveal patterns in outcome data. We reported survival in a standardized form as 1, 3, and 5-year probabilities to survive and calculated change from baseline to follow-up for morbidity and QOL outcomes, in case not reported. In case multiple reports for one study were available, we only reported characteristics or outcomes for the most recent time period and for incident patients, to minimize survival bias (see Supplementary Method 5 for detailed methods).

### Data synthesis

We conducted a narrative synthesis of the results separately by type of outcome and age group and further grouped studies by region, due to geographic variation in healthcare systems and availability of treatments.

We conducted post-hoc meta-analyses to stratify and explore differences in adult survival by relevant subgroups that might explain heterogeneity, including geographic region, disease severity, study period, and study representativeness, whenever there were three or more studies in each subgroup. We operationalized study period as the mid-year of diagnosis or enrollment into the study and used equal-sized subgroups for analyses. We used FC at baseline (ie. proportion of patients with FC III/IV) as a proxy for disease severity with equal-sized subgroups (see Supplementary Method 6 for detailed methods). Subgroup meta-analyses were conducted using random effects models with inverse Freeman-Tukey transformed proportions and the restricted maximum likelihood estimator for between-study variance [33, 34]. Analyses were conducted using Stata 18.0 (Stata Corp, College Station, Texas).

## Results

### Search results

**Fig. 1:**
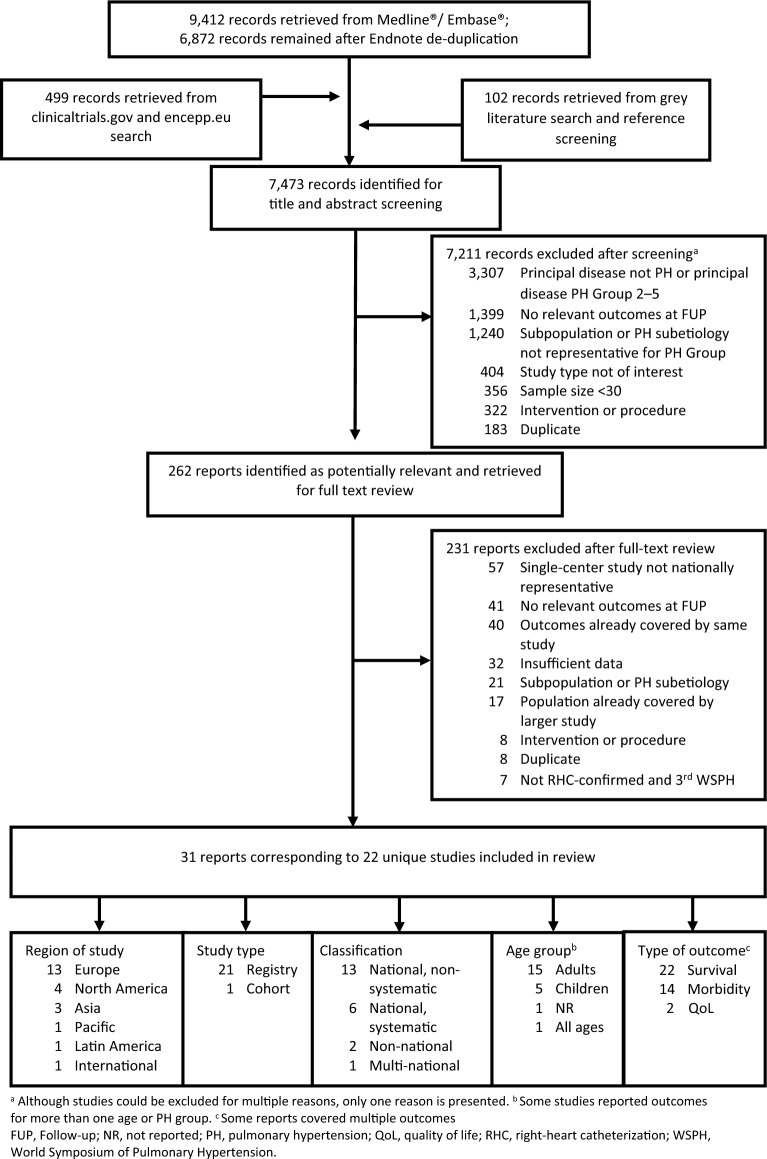
Flow diagram of selection of studies for inclusion into the review **a** Although studies could be excluded for multiple reasons, only one reason is presented. **b** Some studies reported outcomes for more than one age or PH group. **c** Some reports covered multiple outcomes. *FUP* follow-up, *NR* not reported, *PH* pulmonary hypertension, *QoL* quality of life, *RHC* right-heart catheterization, *WSPH* World Symposium of Pulmonary Hypertension.

A flow chart summarizing the study selection is presented in Fig. 1. In total, 7473 records were identified from the searches. Following screening of titles and abstracts, 262 records were selected as potentially relevant. After full text review, 31 reports corresponding to 22 unique population-based studies that provided estimates on survival, morbidity or QoL outcomes were identified and included in the review.

Most studies were from Europe (n = 13), followed by North America (n = 4), Asia (n = 3), Pacific (n = 1), and Latin America (n = 1), see Table 2. Most studies were described as registries (n = 21), mostly (n = 19) with prospective data collection. The majority of the included studies were classified as national, non-systematic (n = 13); while six national, systematic studies, two non-national studies, and one multi-national study were included. Seventeen studies covered adult patients (two were counted as adult as including patients of all ages or not specifying age group) and five studies pediatric PAH patients. All studies reported survival, fourteen studies reported morbidity outcomes, and only two studies reported QOL. Most frequently reported morbidity outcomes across studies were prostacyclin analog use (n = 8) and transplantations (n = 7), followed by risk scores (n = 6), functional class (n = 5), oxygen use (n = 3), hospitalization (n = 3), and 6MWD (n = 3). Clinical worsening and BNP/NT-proBNP were less frequently reported (n = 2 and 1, respectively). QoL outcomes at follow-up were only reported by one adult and one pediatric study.

**Table 2:** Outcomes reported across all population-based studies, by region, age group, and overall

	Survival	Any morbidity	Prostacyclin analog use^a^	Transplantation	Risk score	Functional class	Oxygen	Hospitalization	6MWD	Clinical worsening	BNP/NT-proBNP	QoL
Overall	22	14	8	7	6	5	3	3	3	2	1	2
Region												
Europe	13	9	5	5	3	2	2	2	2	1	-	1
North America	4	3	2	2	2	2	1	1	1	1	1	1
Asia	3	2	1	–	1	1	–	–	–	–	–	–
Pacific	1	–	–	–	–	–	–	–	–	–	–	–
Latin America	1	–	–	–	–	–	–	–	–	–	–	–
Age group												
Adult	17	10	5	5	6	3	3	1	1	1	1	1
Pediatric	5	4	3	2	–	2	–	2	2	1	–	1

Some studies covered multiple outcomes across different reports. ^a^Potentially associated with disease progression

*6MWD* 6-min walk distance, *BNP* brain natriuretic peptide, *NT*-*proBNP* N-terminal pro-brain natriuretic peptide, *QoL* quality of life

### Survival in adults with PAH

Included studies reporting adult survival data (n = 17) were all registries. Four studies were classified as systematic studies, while ten studies were non-systematic and two non-national. Most studies exclusively covered incident patients (n = 15), while one Russian study [35] did not report and one US study included only 52% incident patients [36]. All studies diagnosed patients according to RHC, except for a Korean study where only 36% of patients were diagnosed by RHC and the rest by echocardiography [22]. Majority of studies (n = 14) covered patients with earliest date of diagnosis more than a decade ago (i.e., before 2014), while the latest year of follow-up was 2019 or later for six studies. Global 1-, 3-, and 5-year survival ranged from 85 to 99% (n = 15 studies), from 65 to 95% (n = 14), and from 50 to 86% (n = 9), respectively (See Table 3).

**Table 3:** Survival for adults with PAH by region, study classification, and time period (17 studies/reports)

Study and reference	Classification/ study design	Country	Time period	Participants	Characteristics at baseline				PH Therapy, %	Survival, %		
					Age (yrs)	Female, %	mPAP (mmHg)	FC III/IV, %		1-year	3-year	5- year
Europe												
COMPERA registry [51]	Multi-national, retrospective, prospective, 62 centers	Germany (~ 80%) and 11 other EU countries	2010–2019	Inc (100%) ≥ 18 years n = 2531^a^	65	64	44	84	> 99	90 *	69 *	55 *
Czech National Registry [82]	National, systematic, retrospective, multi-center	Czech Republic	2007–2010	Inc (100%) ≥ 18 yrs n = 91	60 †	66	52 †‡	77 †	92 †	88	74	–
Latvian PH Registry [30]	National, systematic, prospective, single-center	Latvia	2007–2016	Inc (100%) ≥ 18 yrs n = 130	65 †	73	49 †	72 †	99 †	88	73	58
Swedish National PH Registry [83]	National, systematic, retrospective/ prospective, 7 centers	Sweden	2008–2014	Inc (100%) ≥ 18 yrs n = 457	67 †	64	45 †	77 †	100 †	85 *	71 *	59 *
National Audit of PH in Great Britain [84]	National, systematic, prospective, 8 centers	Great Britain (England, Scotland, Wales)	2009–2021	Inc (100%) ≥ 18 yrs n = 5332	61	68	–	–	100^b^	86	65	50
Portuguese Registry [53]	National, non-systematic, prospective, 5 centers	Portugal	2008–2010	Inc (100%) Adults n = 46	43 †‡	65	51 †	71 †	91^c^	94	–	–
Swiss PH Registry [74]	National, non-systematic, retrospective, 13 centers	Switzerland	2016–2019	Inc (100%) > 18 yrs n = 560	–	–	–	–	–	–	95 *	-
French PAH Registry [78]	National, non-systematic, retrospective/ prospective	France	2009–2020	Inc (100%) ≥ 18 yrs n = 2879^d^	61 ‡	60	45	68	79	88 *	69 *	52 *
Russian National Registry [35]	National, non-systematic, retrospective/ prospective, 15 centers	Russia	< 2012–2017	Inc/prev NR > 18 yrs n = 470	43 †‡	84	–	70 †	–	99	94	86
North America												
REVEAL registry [44]	National, non-systematic, retrospective/ prospective, 55 centers	United States	2006–2009	Inc (100%) ≥ 18 yrs n = 710	53 †	78	50	74	95	86	69	61
PH Association Registry [85]	National, non-systematic, retrospective/ prospective, 67 centers	United States	< 2015–2020	Inc (52%) ≥ 18 yrs n = 935	55 ‡	76	–	–	–	92	79	–
PH Connection Registry [54]	Non-national, retrospective/ prospective, 3 centers	United States	2004–2006	Inc (100%) ≥ 18 yrs n = 82	51 ‡	76	51	83	15	85	–	–
Asia												
Japan PH Registry [48]	National, non-systematic, retrospective, 8 centers	Japan	2008–2013	Inc (100%) ≥ 18 yrs n = 108	49 †‡	86	47 †	66 †	100	97 *	88 *	–
Korean Registry of PAH [22]	National, non-systematic, prospective, 35 centers	Korea	2008–2011	Inc (100%) > 18 yrs n = 297 RHC^e^ (36%)	50 ‡	78	52 †	48	61	91	84	–
Japanese Pulmonary Circulation Society Registry [86]	Non-national, retrospective/ prospective, 20 centers	Japan	2012–2016	Inc (100%) Age: - n = 190	49 ‡	77 §	45 §	53 §	92^c^ §	–	85 *	76 *
Pacific												
PH Society of Australia and New Zealand registry [87]	National, non-systematic, prospective, 21 centers	Australia, New Zealand	2011–2019	Inc (100%) ≥ 18 yrs n = 253	58^f^ §	77^f^ §	–	58^f^ §	–	91^f^	–	65^f^
Latin America												
Registry of PH in Argentina [88]	National, non-systematic, prospective, multi-center	Argentina	2014–2016	Inc (100%) > 3 m n = 399	47 ‡§	79 §	52 §	65 §	81 §	93	79	–

Unless otherwise indicated: characteristics and PH-targeted therapy are reported at enrollment, age reported as median, survival based on all-cause deaths

^*^All-cause death and transplant-free; ^†^Reported at diagnosis; ^‡^ Mean; ^§^ Data for entire cohort including incident and prevalent patients; Extracted from graph using Engauge Digitizer;

^a^Two reports included providing survival for adult Group 1 from COMPERA. Selected current reference for synthesis as analysis population unselected, whereas other reference [79] includes only patients with available data for risk score calculation; ^b^Data from cohort of active PH patients of all Groups, subset of survival cohort; ^c^Maximal therapy during follow-up; ^d^A second reference included provided survival for adult Group 1 from French PAH Registry. Selected current reference for synthesis as other reference covers selected subpopulation for risk calculation [46]; ^e^Some patients were diagnosed by echocardiography. ^f^Characteristics are for 1-year post-enrollment and survival from 1-year post-enrollment;

*COMPERA* Comparative, Prospective Registry of Newly Initiated Therapies for Pulmonary Hypertension, *EU* European, *FC* functional class, *inc* incident patients, *m* months, *mPAP* mean pulmonary artery pressure, *NR* *or* *-*, not reported, *PH* pulmonary hypertension, *Prev* prevalent patients, *REVEAL* Registry to Evaluate Early and Long-term PAH Disease Management, *RHC* right-heart catheterization yrs, years

**Fig. 2:**
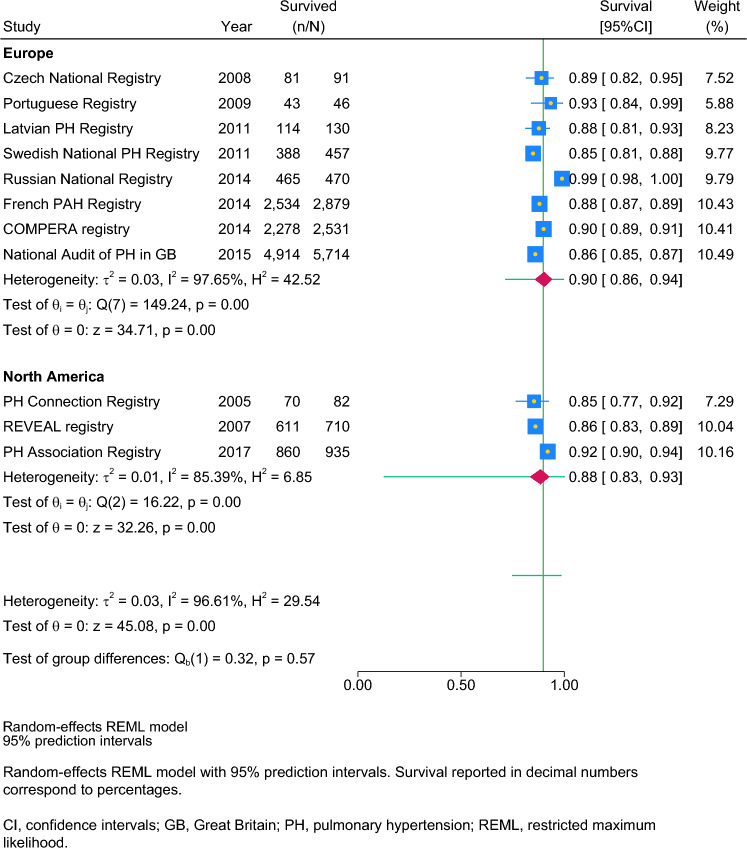
Post-hoc subgroup meta-analysis of adult 1-year survival by region. Random-effects REML model with 95% prediction intervals. Survival reported in decimal numbers correspond to percentages. *CI* confidence intervals, *GB* Great Britain, *PH* pulmonary hypertension, *REML* restricted maximum likelihood

Post-hoc subgroup meta-analysis showed no differences in survival by region. Stratification showed that 1-year survival in Europe was 90% ([95% confidence interval: 86–94%], n = 8 studies pooled, subgroup heterogeneity I^2^: 98%) and in North America 88% ([83–93%], n = 3, I^2^: 85%) (see Table 4 and Fig. 2); 3-year survival in Europe was 78% ([68–86%], n = 8, I^2^: 99%) and in Asia 85% ([82–88%], n = 3, I^2^: 0%, see Table S1, Figure S1); and 5-year survival in Europe was 61% ([49–72%], n = 6, I^2^: 99%, see Table S2, Figure S2).

**Table 4:** Adult 1-year survival by relevant subgroups as assessed by post-hoc meta-analyses

Subgroup	Number of studies	Survival, % [95% CI]	Test for heterogeneity I^2^, %	P for heterogeneity in subgroups
Region of the study^a^				
Europe	8	90 [86–94]	98	p < 0.001
North America	3	88 [83–93]	85	p < 0.001
Baseline functional class III/IV				
47–71%	6	95 [91–98]	85	p < 0.001
72–83%	7	88 [48, 83–85]	67	p = 0.01
Representativeness of the study				
Non-systematic	10	92 [89–95]	93	p < 0.001
Systematic	4	86 [82–84]	0	p = 0.73
Mid-year of diagnosis or enrolment into the study				
2005–2011	8	89 [48, 83–88]	75	p < 0.001
2012–2017	7	92 [88–95]	98	p < 0.001

^a^Three regions were dropped from subgroup analysis due to small group size with survival data available: Asia (n = 2), Latin America (n = 1), and Pacific (n = 1) *CI* confidence intervals

**Fig. 3:**
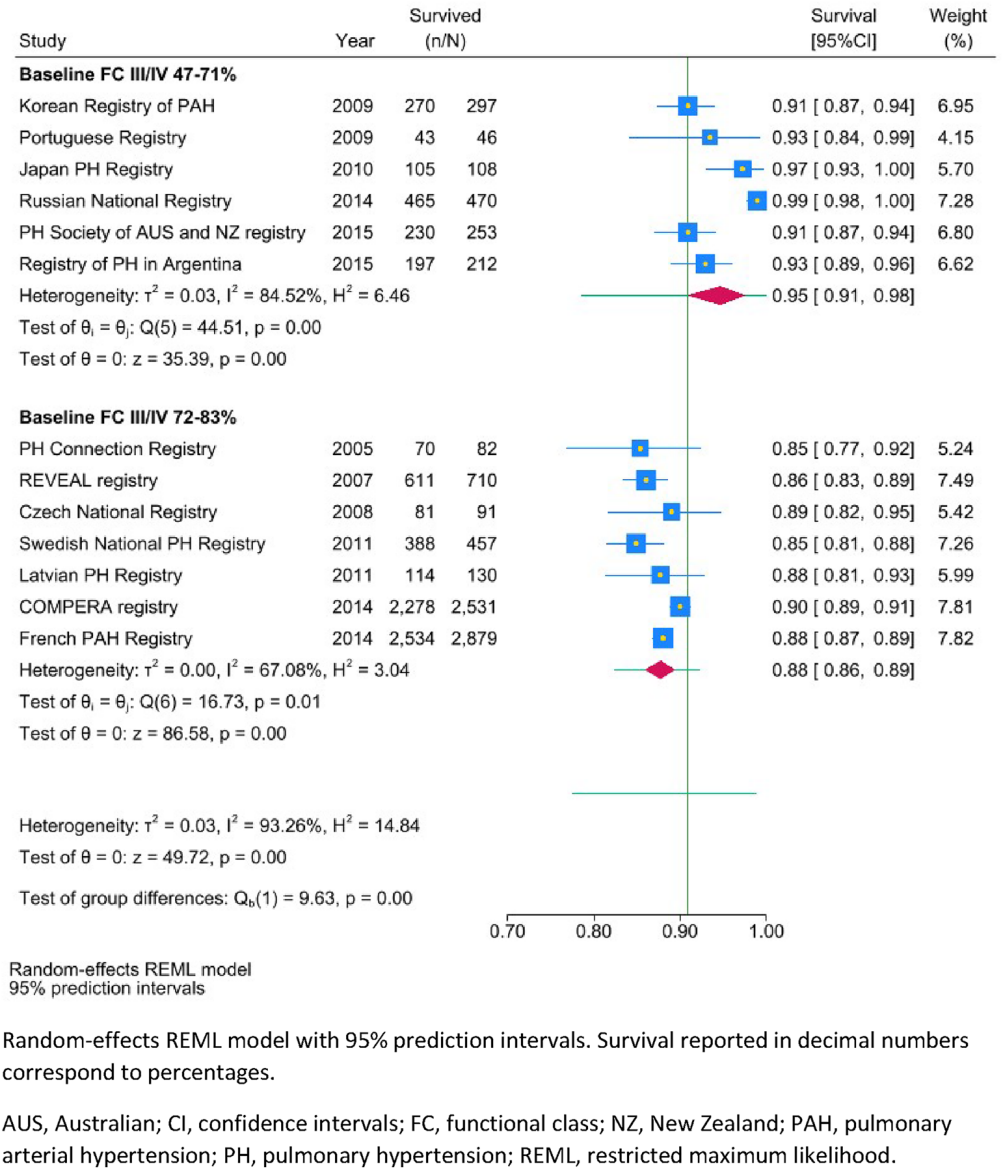
Post-hoc subgroup meta-analysis of adult 1-year survival by baseline functional class. Random-effects REML model with 95% prediction intervals. Survival reported in decimal numbers correspond to percentages. *AUS* Australian, *CI* confidence intervals, *FC* functional class, *NZ* New Zealand, *PAH* pulmonary arterial hypertension, *PH* pulmonary hypertension, *REML* restricted maximum likelihood

We found that survival was higher in studies with a lower proportion of patients with baseline FC III/IV (47–71%) compared to higher proportion of baseline FC III/IV (72 to 83%) for 1-year survival (95% [91–98%], n = 6, I^2^: 85% compared to 88% [86–89%], n = 7, I^2^: 67%, see Table 4, Fig. 3), 3-year survival (87% [81%-91%], n = 5, I^2^: 87% compared to 69% [68–70%]; n = 6; I^2^: 3%, see Table S1, Figure S3), and 5-year survival (76% [63–87%], n = 3, I^2^: 94% compared to 57% [53–60%]; n = 5; I^2^: 84%, see Table S2, Figure S4).

**Fig. 4:**
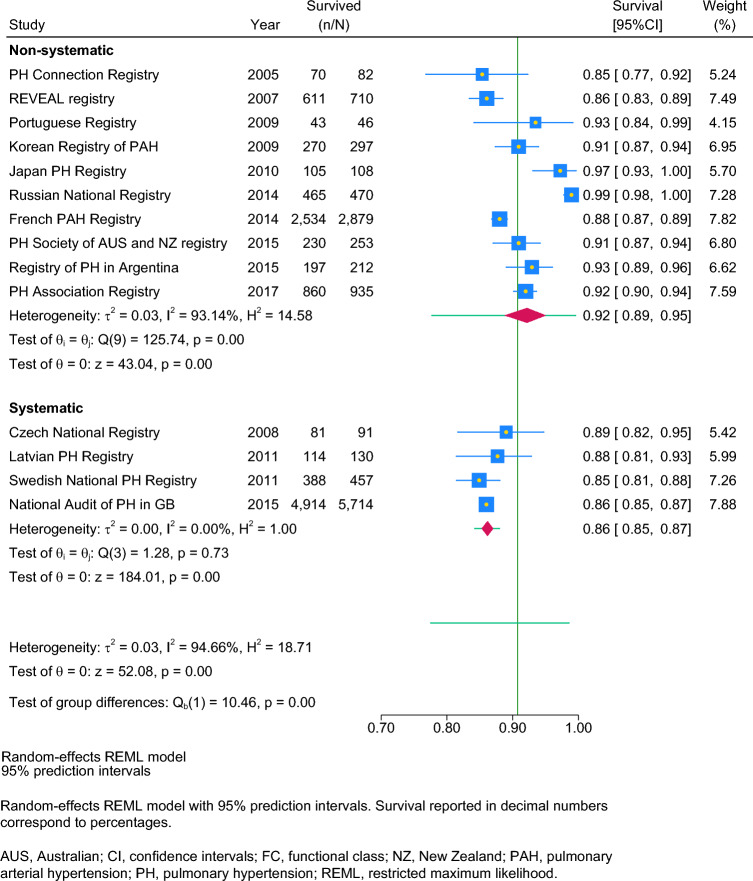
Post-hoc subgroup meta analysis of 1-year survival by study representativeness. Random-effects REML model with 95% prediction intervals. Survival reported in decimal numbers correspond to percentages. *AUS* Australia, *CI* confidence intervals, *GB* Great Britain, *NZ* New Zealand, *PAH* pulmonary arterial hypertension, *PH* pulmonary hypertension, *REML* restricted maximum likelihood

We found higher 1-year survival in studies with non-systematic representativeness (92% [89–95%]; n = 10; I^2^: 93%) compared to studies with systematic representativeness (86% [85–87%], n = 4, I^2^: 0%) (see Table 4, Fig. 4), while no difference were found for 3-year survival (see Table S1, Figure S5) and 5-year survival (see Table S2, Figure S6).

Subgroup analyses showed no differences in 1, 3, or 5-year survival by mid-year of diagnosis/ enrollment into the study, comparing the time periods 2005–2011 to 2012–2017 (see Tables 4, S1-S2, Figures S7-S9).

### Survival in children with PAH

Five studies were included that reported survival data for pediatric PAH patients (See Table 5). All studies were national registries; however, only two had systematic coverage of all centers within their countries. Only two studies reported the percentage of incident patients; the Polish registry of PH reported 13% (n = 80) [37] and the pediatric arm of the US-based REVEAL registry reported 14% (n = 216) [38], while three studies did not provide any information. Exclusive RHC diagnosis was reported by three studies, while a French study [39] and a Spanish study [40] had a coverage of RHC diagnosis of 86% and 95%, respectively. Three registries had earliest patients enrolled more than three decades ago (i.e., before 2003), while earliest diagnosis date was only reported for one registry (1998, Spanish registry) [40]. Only one Polish study enrolled patients up to 2019 [37].

**Table 5:** Survival for pediatric patients with PAH by region, study classification, and time period (5 studies/reports)

Study and reference	Classification/ Study design	Country	Time period	Participants	Characteristics at baseline				PH Therapy, %	Survival, %		
					Age (yrs)	Female, %	mPAP (mmHg)	FC III/IV, %		1-year	3-year	5- year
Europe												
Dutch National Network for Pediatric PH Registry [41]	National, systematic, prospective, 8 centers	Nether-lands	< 2000–2014	Inc/Prev NR Children n = 70	8	66	54	59	100	76 *	64 *	56 *
Polish Registry of PH [37]	National, systematic, retrospective/ prospective, 8 centers	Poland	< 2018–2019	Inc (13%) ≥ 3 m to ≤ 18yrs n = 80	6 †	50 ‡	48 ‡§	31 ‡	98 ‡	98 ‡¶	–	–
French pediatric PAH Registry [39]	National, non-systematic, retrospective/ prospective, 21 centers	France	< 2005–2008	Inc/Prev NR ≥ 28d to ≤ 18yrs n = 50 RHC^a^ (86%)	9 †‡	48 ‡	59 ‡	28 ‡	82 ‡	86 ‡	–	–
Spanish Registry for Pediatric PH [40]	National, non-systematic, retrospective/ prospective, 21 centers	Spain	1998–2012	Inc/Prev NR ≥ 2 m to ≤ 18yrs n = 142 RHC^a^ (95%)	5 †	55	46 ‡§	51 ‡§	-	89 ‡¶	85 ‡¶	–
North America												
REVEAL registry – pediatric arm [38]	National, non-systematic, retrospective/ prospective, 26 centers	US	< 1995–2010	Inc (14%) ≥ 3 months to ≤ 18 years n = 216	15	64	56 §	28	74	96	84	74

Unless otherwise indicated: characteristics and PH-targeted therapy are reported at enrollment, age reported as median, survival based on all-cause deaths

^*^All-cause death and transplant-free; ^†^ Mean; ^‡^Data for entire cohort including incident and prevalent patients; ^§^Reported at diagnosis;

Extracted from graph using Engauge Digitizer; ^a^Some patients were diagnosed by echocardiography

*FC* functional class, *inc* incident patients, *m* months, *mPAP* mean pulmonary artery pressure, *NR* or *-* not reported, *PAH* pulmonary arterial hypertension, *PH* pulmonary hypertension, *Prev* prevalent patients, *REVEAL* Registry to Evaluate Early and Long-term PAH Disease Management, *RHC* right-heart catheterization, *yrs* years

**Fig. 5:**
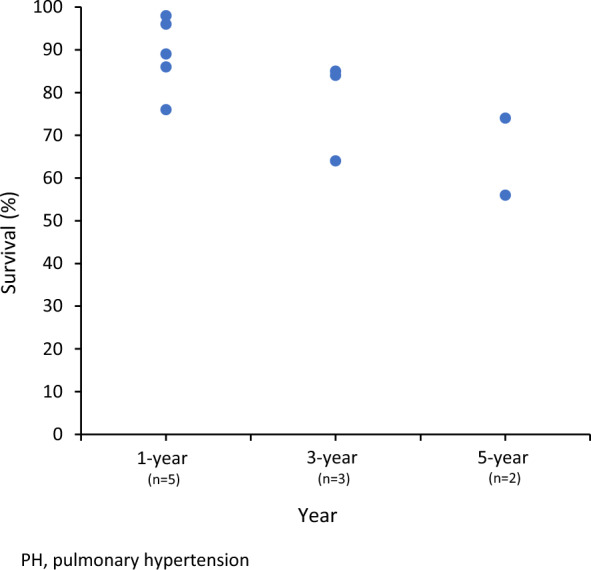
Global 1- 3-, and 5-year survival of pediatric PH Group 1 patients PH, pulmonary hypertension

Across all five studies, 1- and 3-year survival ranged from 76 to 98% (n = 5) and 64 to 85% (n = 3). 5-year survival was 56% in a Dutch study [41] and 74% in a US study [38], summarized in Fig. 5.

### Morbidity and QoL in adults with PAH

Ten studies were included that reported morbidity or QoL outcomes for adult PAH, based on 15 individual reports as three studies covered multiple reports on relevant outcomes (Swedish National PH Registry [42, 43], REVEAL registry [20, 44–46], and Japan PH Registry [47, 48]) (See Table S3). All studies reported morbidity outcomes, whereas only one study [49] reported QoL outcomes with available follow-up estimates. All studies were described as registries; only two had national systematic coverage. Six studies reported risk scores, mostly describing the development or validation of an ESC/ERS-based risk score calculator. Changes in risk scores were available from four studies, with improvements between 20 to 59% after a median/mean follow-up of 4 to 8 months. Five studies reported lung transplantation, ranging between 0.8% and 3.9% in patients after a median/mean follow-up of 31 to 46 months [30, 43, 45, 50, 51]. Prostacyclin analog use at follow-up was reported by five studies, and estimates of change were available from three studies [42, 49, 52], ranging from −2% to + 22% across all follow-up timepoints. Three studies covered WHO or New York Heart Association (NYHA) FC, of which two reported a worsening in FC III/IV at follow-up of −9 [49] to −24% [48] after a median/mean follow-up of 8 to 10 months and one largely unchanged FC (63%) after follow-up within 12 months [44]. Oxygen use at follow-up was reported by three studies [42, 49, 53], with different follow-up duration and follow-up time points. Outcomes only reported by one study each included BNP [45], hospitalization and QoL [49]. Borgese et al. [49] reported QoL via utilization of the EmPHasis-10 (e10) questionnaire. The authors reported a mean e10 score of 25 at baseline and found that scores were lower at subsequent follow-up visits; the greatest decline was by five points at third follow-up, representing a notable decrease in QoL (median 10 months between each follow-up visit).

### Morbidity and QoL in children with PAH

Four studies were included that reported morbidity or QoL outcomes for pediatric PAH patients (See Table S4). All studies were national registry studies but only two had systematic coverage of all centers in their countries of origin. Three registries reported proportion of incident patients, which were 12% in the Polish registry, 14% in US registry, and 30% in the French registry [37, 39, 54]. The most frequently reported morbidity outcome in pediatric patients was prostacyclin analog use. Three studies reported changes in prostacyclin analog use, ranging from -4 to 18% over a median/mean follow-up of 17 to 23 months [37, 39, 41]. Other morbidity outcomes were reported less frequently at follow-up: hospitalization (n = 2), transplantation (n = 2), WHO FC (n = 2), and 6MWD (n = 2). QoL was only reported by a French study [39], who used the Child Health Questionnaire—Parent Form 50 (CHQ-PF50). Risk scores, oxygen use, and BNP were not reported at follow-up by any of the identified studies.

## Discussion

To our knowledge, this is the first systematic literature review to report current real-world survival, morbidity, and QoL outcomes for RHC-confirmed PAH in adult and pediatric patients. We aimed to describe disease outcomes based on 3rd WSPH Venice classification or later, that are most comparable and representative at the population-level. Most studies were prospective registries from Europe or North America, with few outcome data available for other regions. Although most studies identified were disease registries aiming to describe generalizable characteristics and disease history, only few were truly representative at the national level. Survival was the most frequently reported outcome. Post-hoc subgroup meta-analyses revealed that better survival in adult PAH was consistently associated with better baseline FC. No differences in survival by region or study period were found; differences by study representativeness were only found for 1-year survival. Few studies reported morbidity or QoL estimates, with risk scores at follow-up mainly reported for adult PAH. Overall, most outcome data were available for adult patients, while comparable and population-based disease outcomes for pediatric patients were scarce – the few that exist are mostly from Europe and cover prevalent patients dating back more than three decades.

### Representativeness of studies

Most studies that matched stringent eligibility criteria were large disease registries. Since the principal goal of these registries is to characterize and describe the natural history with the aim to improve prognosis for current and future patients, generalizability of their findings is of central importance [16, 31, 55, 56]. While most studies were described as ‘national’ studies by authors, only six studies were assessed as systematically covering and enrolling patients from all expert centers in their country of origin.

We did not find consistent differences across all survival metrics between studies that were truly representative at the population level compared to those that were not, which seems plausible as we do not expect that representativeness has a particularly strong effect with a clear direction on survival. Nevertheless, we expect that survival estimates provided by the six systematic registries provide the most accurate representation of participant characteristics and survival in their respective countries.

### Origin of studies

Most studies reviewed originated from Europe or North America, with few outcome data available for low-to-middle-income countries (LMICs), suggesting a lack of comparable outcome data reported by population-based studies from LMICs. This is important to highlight, given that the global burden of disease of overall PH lies disproportionately in the developing world [57], due to higher prevalence of underlying diseases (e.g., HIV/AIDS, schistosomiasis) as well as environmental factors (e.g., high altitude, air pollution) [58]. Despite lack of resources and limited PH-related expertise in LMICs [59], a small number of registries exist, such as the African PAPUCO registry [60], the Indian PRO-KELARA registry [61], or the Ukrainian registry [62]. These and other registries were not eligible for inclusion into the review as they often only exclusively used echocardiography for diagnosis, had no relevant outcomes published at follow-up or were small single-center studies. Further studies of PAH outcomes in LMICs are required to better understand the prognosis of PAH in these under-represented regions.

Our subgroup analysis did not show differences in adult survival when compared across regions. This was surprising, as survival differences across regions and especially between Western countries and LMICs seem a plausible assumption, due to different healthcare systems, diagnostic procedures, and treatment strategies. However, low number of comparator studies from Asia or Latin America precludes drawing sound conclusions and an actual effect could be confounded by other factors such as study period, baseline disease severity, or other characteristics of patients enrolled in the studies.

### Pediatric patients

Comparable and population-based survival data for pediatric PAH were scarce, as only five registries were identified and included in this review, mostly including prevalent patients with diagnosis dates from up to three decades ago. Identified survival estimates are therefore likely poorly generalizable to newly diagnosed patients of the current treatment era. Low prevalence of pediatric compared to adult PAH (4 to 14 compared to 48 to 55 cases per million in Europe) [2] and ethical and logistical difficulties [63, 64] likely limit the availability of participants in pediatric research and might explain scarcity of studies. Furthermore, due to associated risks with RHC [65–67], young children are often diagnosed based on non-invasive diagnostic procedures such as echocardiography. While we did not restrict inclusion of studies exclusively to RHC diagnoses and thus also cover patient populations diagnosed by both RHC and echocardiography, we might have excluded pediatric studies that exclusively covered patients diagnosed based on non-invasive procedures.

After our search cut-off date in November 2021, findings of three large registries on pediatric patients with PH were published: the Tracking Outcomes and Practice in Pediatric Pulmonary Hypertension registry covering 20 countries worldwide conducted between 2008 and 2015 with n = 242 PAH patients enrolled [68], the US Pediatric Pulmonary Hypertension Network conducted between 2001 and 2021 with n = 602 PAH patients [69] and the UK National Pediatric Pulmonary Hypertension Service conducted between 2001 and 2021 with n = 529 PAH patients [70]. Both registries enrolled children based on invasive or non-invasive diagnosis and provide important insights on current characteristics and survival of pediatric patients with PAH. The more recent data captured and the large number of patients enrolled in these registries will provide very important information in pediatric patients in coming years. Nevertheless, we still believe that further up-to-date pediatric PAH registries are needed to better inform the management and treatment of children [71], especially from other regions than Europe or North America.

### Survival

Across all studies identified, survival was the most commonly reported outcome. This is not surprising, as the ultimate aim of PAH treatment goal is to achieve prolonged survival [17]. While our post-hoc meta-analysis did not find differences in survival across regions and findings were inconsistent for representativeness as discussed earlier, we observed higher survival in studies with lower proportion of FC III/IV patients, indicative of lower disease severity at baseline. This finding, consistent across subgroup analyses of 1-, 3-, and 5-year survival, is plausible and consistent with the wide usage of FC to assess disease severity and predict survival of patients with PAH. [20]

We did not observe differences in survival by study period when comparing studies with mid-diagnosis/ enrolment year between 2005 and 2011 to those between 2012 and 2017, despite availability of additional therapies and risk stratification strategies for PAH in the latter period. This finding was consistent with analyses of registry data from Germany (2010–2019), Canada (2009–2021) and the Netherlands (2005–2009), not finding differences in survival when stratifying by diagnostic period [51, 72, 73]. Authors explained their findings by lack of coverage of combination therapy [51], while others detected an increase in combination therapy that they hypothesized to be overall insufficient [73]. In addition, patients who were diagnosed before publication of the 2015 ESC/ERS guidelines [17] may have escalated to combination therapy after release of the guideline, hence improving survival and narrowing the difference in survival from those who were diagnosed after publication of the guideline [5]. On the other hand, an analysis of the Swiss registry by Appenzeller et al. [74] found improvements in 3-year survival between diagnostic periods 2001–2005 (63%) and 2016- 2019 (95%). In their systematic review, Emmons-Bell et al. [24] reported 1-year survival of patients with PAH across 58 studies to be lower in those published before 1998 compared to after 1998; a finding which however needs to be interpreted with caution as discussed previously.

Our subgroup meta-analysis has important limitations and our finding regarding lack of difference of survival by study period needs to be interpreted with caution due to several reasons. Firstly, heterogeneity remained high across subgroups, possible indicating the presence of other confounding factors that we did not account for. These could include patient characteristics, such as age, gender, and disease severity; as well as heterogeneity across studies, including dropout rate, pace of enrolment, adoption of combination therapy, geography, and differences in real-world practices. Secondly, we chose mid-diagnosis/enrollment year as the proxy for study period, calculated as the median between earliest year of diagnosis/enrollment and end of follow-up of the study. While this might reflect the year of diagnosis most accurately for the majority of the study cohort, it might be a less reliable surrogate for era across studies and thus possibly attenuating the correlation, especially when differences in diagnostic, recruitment, and follow-up periods are pronounced across studies. Thirdly, we chose equal-sized subgroups resulting in arbitrary time periods; and studies with mid-enrolment year between 2012 and 2017 may have significant number of patients treated with monotherapy using previously available PAH medications, therefore not reflecting survival benefits of the newly available PAH therapies in the latter era. Fourthly, reporting of disease severity might be incomplete and meta-analysis results might be impacted if FC data are not missing at random. Given that improvement in survival is the ultimate goal of management of patients with PAH, it is very important to evaluate whether the recent advances in PAH therapies and risk assessment strategies bring about survival improvement in the real world. We encourage investigators from large registries with long follow-up to conduct further intra-study comparisons to further elucidate the issue of changes in survival over time.

### Morbidity and QoL outcomes

Fewer data were available for morbidity outcomes, with prostacyclin analog use, transplantation events, risk score, and FC most frequently reported. While measures of disease severity such as FC and 6MWD are common non-invasive clinical trial endpoints [75, 76], these measures were not often reported in studies included in this review. Scarcity of morbidity outcome data is likely explained by the observational nature of registries that, unlike interventional clinical trials, typically do not mandate clinical follow-up at defined intervals with specific assessments.

Comprehensive assessment of patients’ risk of deterioration based on multiple prognostic predictors is recommended to be conducted periodically as per current guidelines [5]. In our review, we identified five registries that utilized three different risk stratification methods in adult patients (including COMPERA 2.0 3- and 4-risk strata scores [51], ESC/ERS 2015 risk score [43], and REVEAL risk score [77]) and reported estimates at follow-up, mostly as part of development of validation of risk tools [42, 78, 79]. While the risk status of most patients seems to remain unchanged or improved during short-term follow-up within one year, detailed comparison is hampered by the heterogeneity of scoring algorithms, on top of the general difficulties of comparison of morbidity outcomes related to differential duration of observation, timepoints of assessment, and selection bias due to missing data.

Among the few pediatric studies that we identified and reviewed, we also found that morbidity outcomes were scarce. Those that were reported most frequently at follow-up included prostacyclin analog use, hospitalization, transplantation, and WHO FC. Despite risk stratification also being recommended in pediatric patients [5], no data on risk assessment at follow-up was reported by pediatric registries identified. This is in line with a recent systematic literature review on risk assessment tools in PAH, which reports only two studies that used risk stratification in pediatric PAH patients with only one tool existing that was specifically developed for use in children [80], indicating an urgent need for further research in this area.

Only two studies reported QoL (EmPHasis-10 [49] in adult patients and CHQ-PF50 [39] in pediatric patients) as an outcome with estimates at follow-up. Many studies using patient-reported outcomes (PRO) or QoL tools have less of a clinical focus and do not routinely report details regarding diagnosis, are cross-sectional, or based on a single center and were therefore not included in this review. Regardless of that, our findings suggest that QoL outcomes do not seem a priority in large population-based registries. Current ESC/ERS guidelines [5] support our finding, suggesting that PRO and QoL are underused as outcome measures, despite being an important factor in reflecting the symptoms and needs of patients [25]. Routine use and more frequent reporting of PRO and QoL outcome measures during patient follow-up in larger registries or cohort studies is therefore suggested.

### Strengths & limitations

To our knowledge, this is the first systematic review providing a comprehensive overview of current estimates for survival, morbidity, and QoL outcomes for adult and pediatric PH Group 1 based on population-based studies, RHC diagnosis, and revised 3rd WSPH classification or later. We employed a rigorous search strategy and used stringent criteria for selection of studies into this review to ensure comparability across studies through homogenous and well-defined patient populations, as well as representativeness of estimates. However, narrow selection criteria came at the cost of potentially introducing selection bias. Only including studies with exclusive or at least partial RHC diagnoses meant likely missing studies from low-resource settings with poor access to RHC and studies covering certain outcomes, in particular QoL. We defined ‘population-based studies’ as those with a minimum level of population representativeness, operationalized through studies being either multi-center (assuming that more than one center covers a larger catchment area and represents patients subject to different clinical practices) or single-center, but with national representativeness. Besides difficulties ascertaining this even after full text review, these criteria might have led to the exclusion of several large (single) referral center studies with very well-defined patient cohorts.

While our eligibility criteria ensured greater comparability of outcomes between included studies, heterogeneity was still encountered when reporting and synthesizing data. Authors used and defined different timepoints to measure or report patients’ characteristics and outcomes. Prospective studies often defined baseline as the time of enrollment, while retrospective studies rather used time-of-diagnosis or did not provide further definition of baseline. To minimize heterogeneity, we reported baseline characteristics at enrollment wherever possible. We reported outcomes based on incident patients whenever available in an attempt to minimize survival bias associated with prevalent patients, presenting a key strength of this review.

Finally, we did not conduct a formal risk of bias assessment due to lack of tailored tools for the study types targeted in this review, being mostly disease registries. While the principal aim of this review was to narratively describe outcomes, we conducted post-hoc subgroup meta analyses to explore differences in survival by relevant subgroups. To confirm our findings and address important limitations of this approach, we recommend conducting a formal meta regression analysis – a more robust approach when analyzing continuous variables which also enables accounting for confounding.

## Conclusion

This systematic review highlights that survival in RHC-confirmed adult patients with PAH is well reported by population-based observational studies, whilst there is a general lack of morbidity and QoL outcomes despite their predictive value and relevance to patients. Outcome data for PAH in children is scarce and the few available estimates are not generalizable to the current treatment era, highlighting the need for up-to-date prospective PAH registries in children. Most identified registries are from Western countries, highlighting the need for comparable and population-based outcome data from regions such as Asia, Latin America, and the Pacific. No differences in survival between regions or over time were observed. Further advances in therapeutic development and management are required to improve long-term prognosis of PAH patients.

## Supplementary Information

Supplementary File 1.

## Data Availability

No datasets were generated or analysed during the current study.

## Abbreviations

6MWD: 6-Min walk distance
BNP: Brain natriuretic peptide
CHQ-PF50: Child Health Questionnaire Parent Form 50
COMPERA: Comparative, prospective registry of newly initiated therapies for pulmonary hypertension
e10: EmPHasis-10
ESC/ERS: European Society of Cardiology and the European Respiratory Society
FC: Functional class
FDA: Food and Drug Administration
FUP: Follow-up
LMIC: Low-to-middle-income countries
mPAP: Mean pulmonary arterial pressure
NT-proBNP: N-terminal fragment of proBNP
NYHA: New York Heart Association
PAWP: Pulmonary arterial wedge pressure
PAH: Pulmonary arterial hypertension
PH: Pulmonary hypertension
PICO: Population, Intervention, Comparator and Outcome
PRO: Patient-reported outcomes
PVR: Pulmonary vascular resistance
QoL: Quality of life
REVEAL: Registry to Evaluate Early and Long-term PAH disease management
RHC: Right-heart catheterization
WSPH: World Symposium of Pulmonary Hypertension
WHO: World Health Organization
WU: Wood Units
