# Supplementary material for: Survival, morbidity, and quality of life in pulmonary arterial hypertension patients: a systematic review of outcomes reported by population-based observational studies
Source: Respir Res. 2024 Oct 16;25:373. doi: 10.1186/s12931-024-02994-w (PMC11481430; doi:10.1186/s12931-024-02994-w)
Supplement: Supplementary file 1 [file 12931_2024_2994_MOESM1_ESM.docx]

## Figure S1: Post-hoc subgroup meta-analysis of adult 3-year survival by region


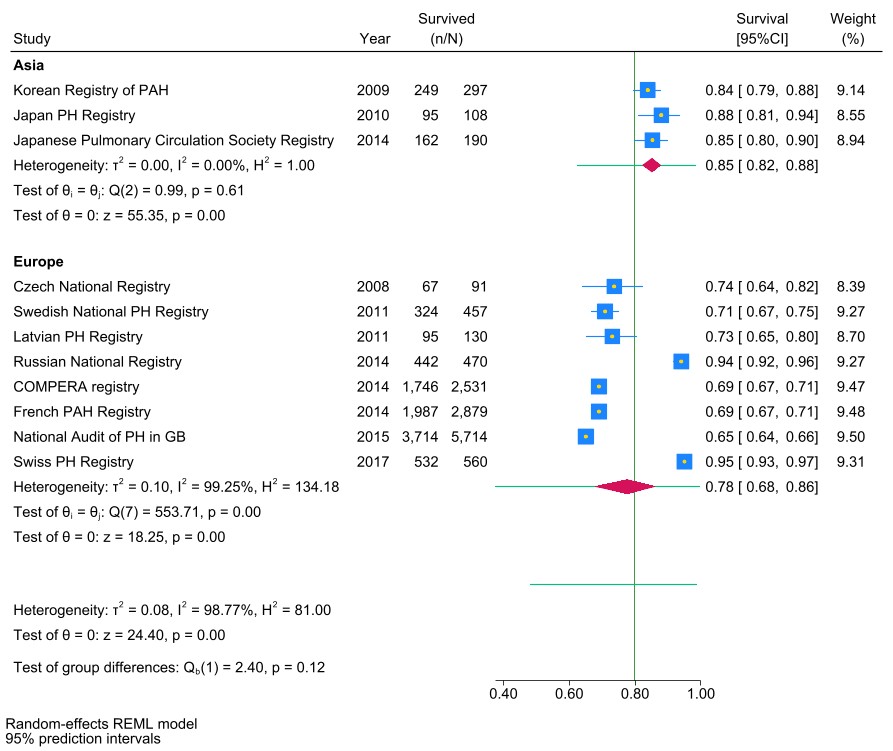


Random-effects REML model with 95% prediction intervals. Survival reported in decimal numbers correspond to percentages.

CI, confidence intervals; GB, Great Britain; PAH, pulmonary arterial hypertension; PH, pulmonary hypertension; REML, restricted maximum likelihood.

## Figure S2 Post-hoc subgroup meta-analysis of adult 5-year survival by region


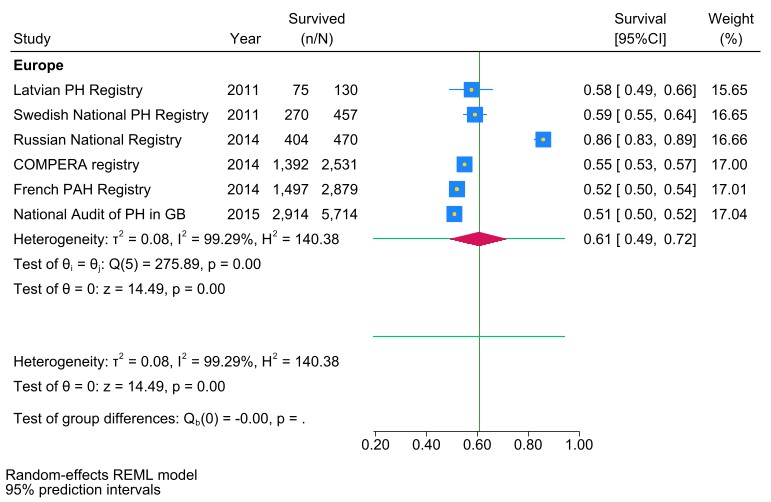


Random-effects REML model with 95% prediction intervals. Survival reported in decimal numbers correspond to percentages.

CI, confidence intervals; GB, Great Britain; PAH, pulmonary arterial hypertension; PH, pulmonary hypertension; REML, restricted maximum likelihood.

## Figure S3: Post-hoc subgroup meta-analysis of adult 3-year survival by baseline functional class


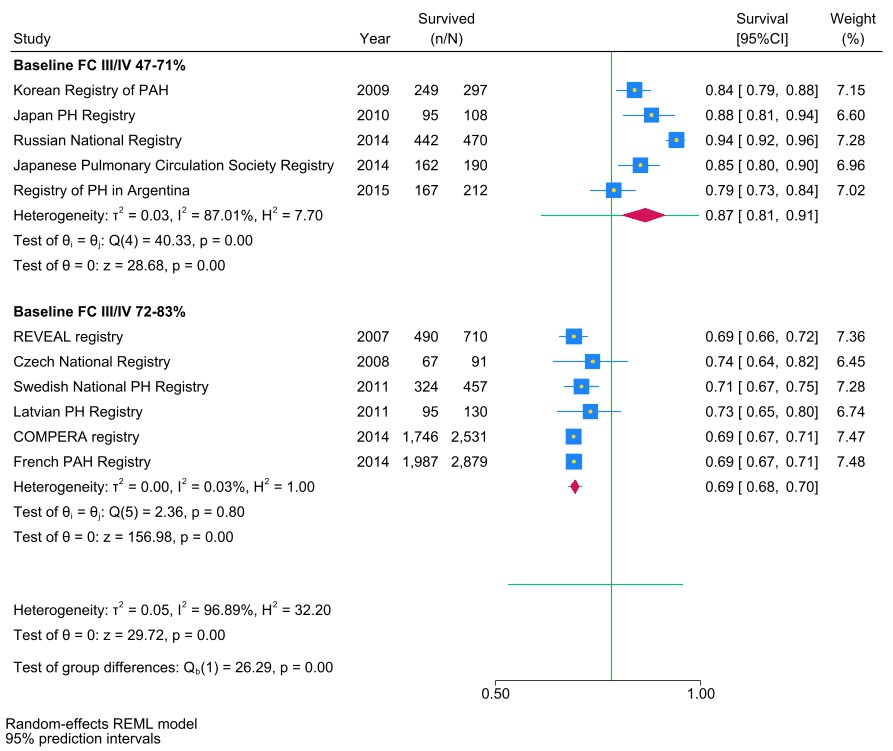


Random-effects REML model with 95% prediction intervals. Survival reported in decimal numbers correspond to percentages.

CI, confidence intervals; PAH, pulmonary arterial hypertension; PH, pulmonary hypertension; REML, restricted maximum likelihood.

## Figure S4: Post-hoc subgroup meta-analysis of adult 5-year survival by baseline functional class


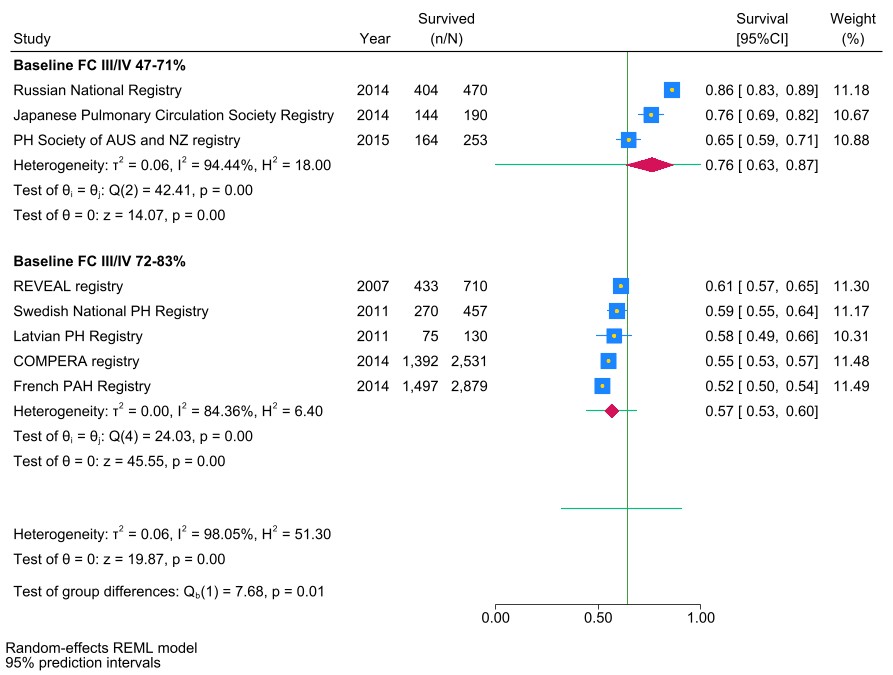


Random-effects REML model with 95% prediction intervals. Survival reported in decimal numbers correspond to percentages.

AUS, Australia; CI, confidence intervals; NZ, New Zealand; PAH, pulmonary arterial hypertension; PH, pulmonary hypertension; REML, restricted maximum likelihood.

## Figure S5: Post-hoc subgroup meta-analysis of adult 3-year survival by classification


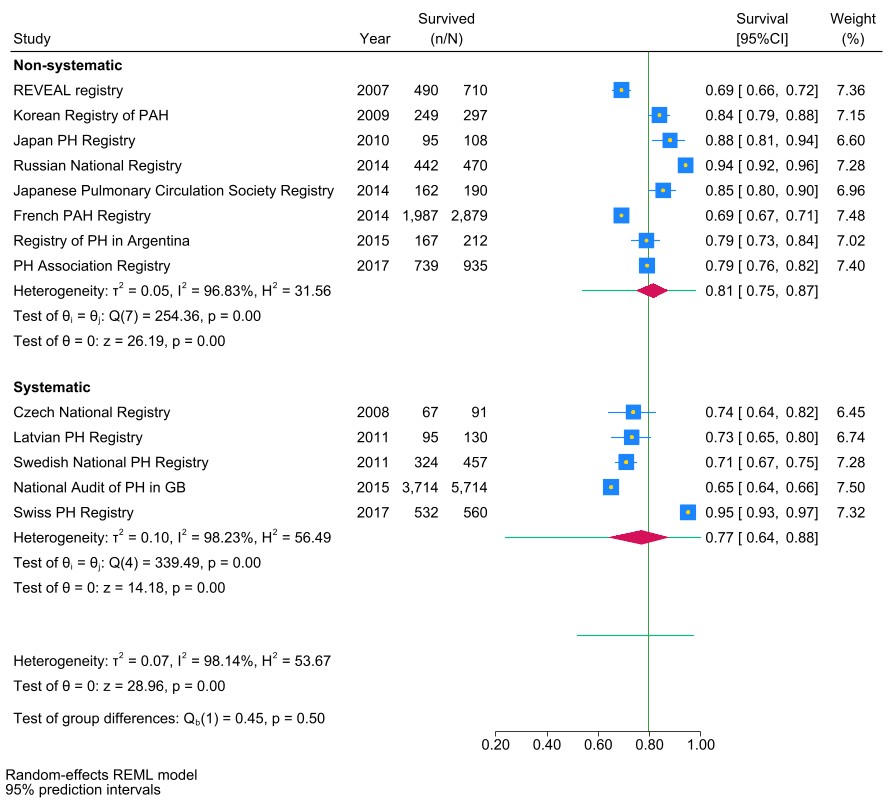


Random-effects REML model with 95% prediction intervals. Survival reported in decimal numbers correspond to percentages.

CI, confidence intervals; GB, Great Britain; PAH, pulmonary arterial hypertension; PH, pulmonary hypertension; REML, restricted maximum likelihood.

## Figure S6: Post-hoc subgroup meta-analysis of adult 5-year survival by classification


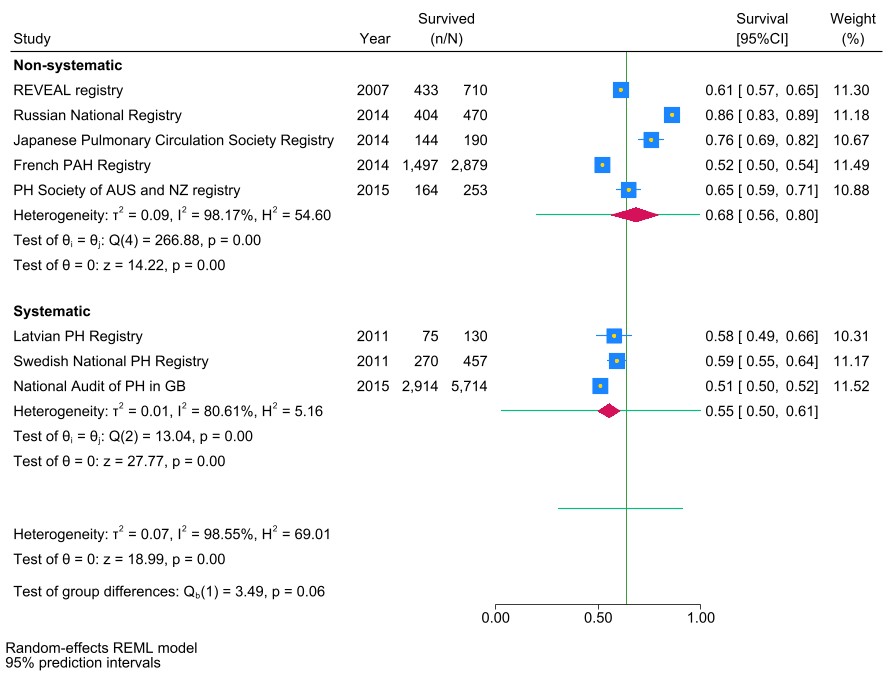


Random-effects REML model with 95% prediction intervals. Survival reported in decimal numbers correspond to percentages.

AUS, Australia; CI, confidence intervals; GB, Great Britain; NZ, New Zealand; PAH, pulmonary arterial hypertension; PH, pulmonary hypertension; REML, restricted maximum likelihood.

## Figure S7: Post-hoc subgroup meta-analysis of adult 1-year survival by study period
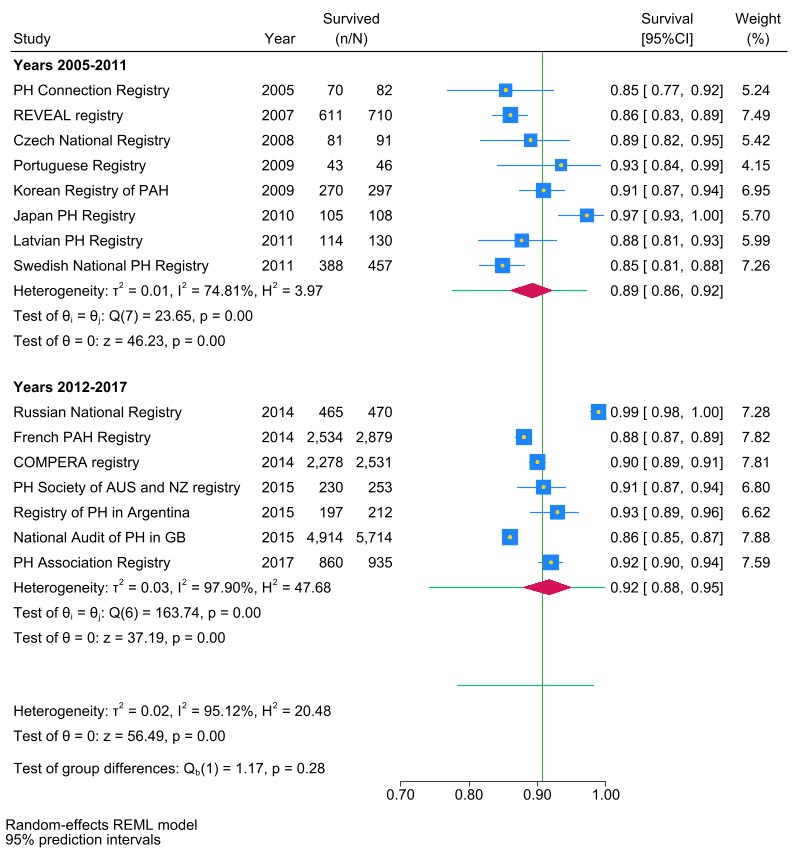


Random-effects REML model with 95% prediction intervals. Survival reported in decimal numbers correspond to percentages.

AUS, Australia; CI, confidence intervals; GB, Great Britain; NZ, New Zealand; PAH, pulmonary arterial hypertension; PH, pulmonary hypertension; REML, restricted maximum likelihood.

## Figure S8: Post-hoc subgroup meta-analysis of adult 3-year survival by study period


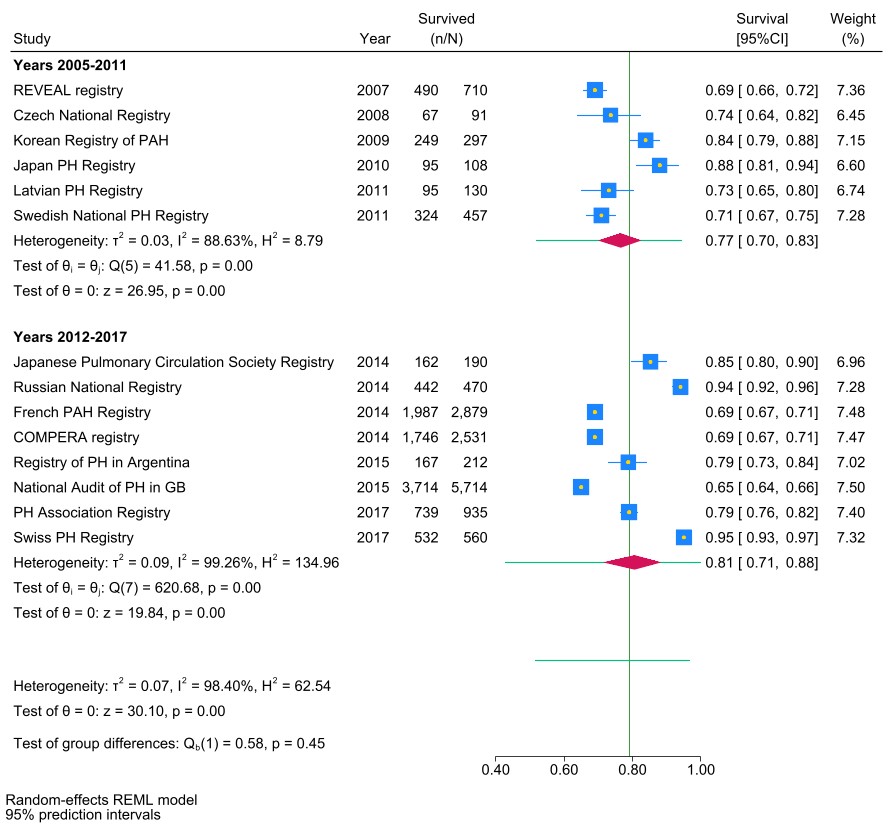


Random-effects REML model with 95% prediction intervals. Survival reported in decimal numbers correspond to percentages.

CI, confidence intervals; GB, Great Britain; PAH, pulmonary arterial hypertension; PH, pulmonary hypertension; REML, restricted maximum likelihood.

## Figure S9: Post-hoc subgroup meta-analysis of adult 5-year survival by study period


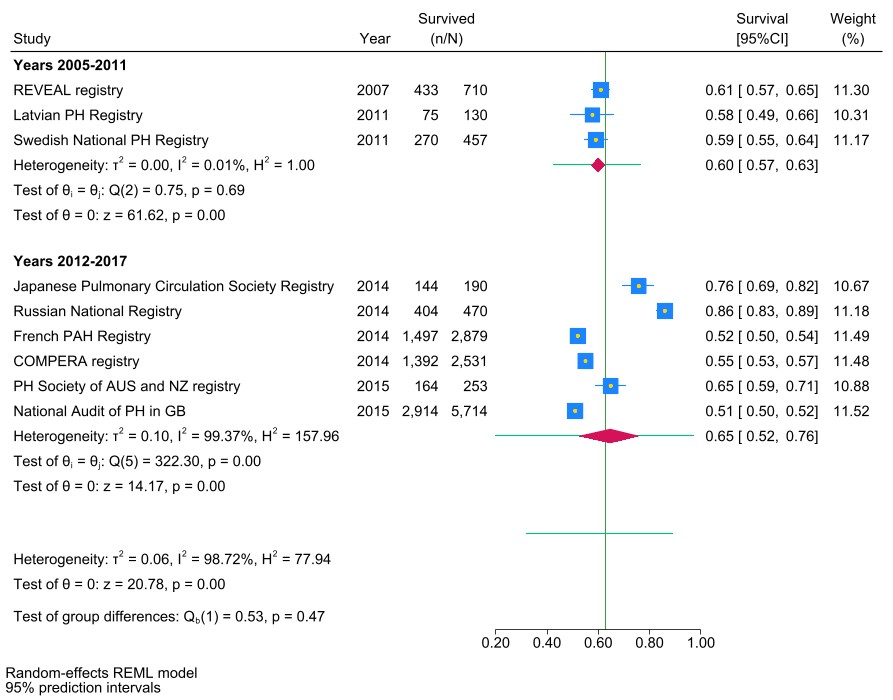


Random-effects REML model with 95% prediction intervals. Survival reported in decimal numbers correspond to percentages.

AUS, Australia; CI, confidence intervals; GB, Great Britain; NZ, New Zealand; PAH, pulmonary arterial hypertension; PH, pulmonary hypertension; REML, restricted maximum likelihood.

## Table S1. Adult 3-year survival by relevant subgroups as assessed by post-hoc meta-analyses

| Subgroup | Number of studies | Survival, %  [95% CI] | Test for heterogeneity I², % | P for heterogeneity in subgroups |
| --- | --- | --- | --- | --- |
| **Region of the study** ^a^ | | | | |
| Europe | 8 | 78 [68-86] | 99 | p<0.001 |
| Asia | 3 | 85 [82-88] | 0 | p=0.61 |
| **Baseline Functional Class III/IV** | | | | |
| 47-71% | 5 | 87 [81-91] | 87 | p<0.001 |
| 72-83% | 6 | 69 [68-70] | 3 | p=0.80 |
| **Representativeness of the study** | | | | |
| Systematic | 5 | 77 [64-88] | 98 | p<0.001 |
| Non-systematic | 8 | 81 [75-97] | 97 | p<0.001 |
| **Mid-year of diagnosis or enrollment into the study** | | | | |
| 2005-2011 | 6 | 77 [70-83] | 87 | p<0.001 |
| 2012-2017 | 8 | 81 [71-88] | 99 | p<0.001 |
| ^a^ Two regions were dropped from subgroup analysis due to small group size with survival data available: North America (n=2), Latin America (n=1); No 3-year survival data available for Pacific (n=1) | | | | |

## Table S2. Adult 5-year survival by relevant subgroups as assessed by post-hoc meta-analyses

| Subgroup | Number of studies | Survival, %  [95% CI] | Test for heterogeneity I², % | P for heterogeneity in subgroups |
| --- | --- | --- | --- | --- |
| **Region of the study** ^a^ | | | | |
| Europe | 6 | 61 [49-72] | 99 | p<0.001 |
| **Baseline Functional Class III/IV** | | | | |
| 47-71% | 3 | 76 [63-87] | 94 | p<0.001 |
| 72-83% | 5 | 57 [53-60] | 84 | p<0.001 |
| **Representativeness of the study** | | | | |
| Systematic | 3 | 55 [50-61] | 81 | p<0.001 |
| Non-systematic | 5 | 68 [56-80] | 98 | p<0.001 |
| **Mid-year of diagnosis or enrolment into the study** | | | | |
| 2005-2011 | 3 | 60 [57-63] | 0 | p=0.69 |
| 2012-2017 | 6 | 65 [52-76] | 99 | p<0.001 |
| ^a^ Three regions were dropped from subgroup analysis due to small group size with survival data available: Asia (n=1), North America (n=1), and Pacific (n=1). No 5-year survival data available for Latin America (n=1) | | | | |

## Table S3. Morbidity and QoL in adult PH Group 1 patients (10 studies, 15 reports)

| **Study and Reference** | **Classification/ Study design** | **Country** | **Time period** | **Participants** | **Characteristics at baseline** | | | | **PH Therapy, %** | **Follow-up duration (months)** | **Morbidity and QoL Outcomes** | | |  |
| --- | --- | --- | --- | --- | --- | --- | --- | --- | --- | --- | --- | --- | --- | --- |
|  |  |  |  |  | **Age (yrs)** | **Female, %** | **mPAP (mmHg)** | **FC III/IV, %** |  |  | **At baseline** | **At follow-up** | **Change** |  |
| **Europe** | | | | | | | | | | | | | | |
| COMPERA [[11](#_ENREF_11)] | Multi-national, retrospective/ prospective registry,  62 centres | Germany (~80%) and 11 other EU countries | 2009-2020 | Inc (100%)  ≥ 18 years  *n* = 1,655 | 66 * | 64 | 43 | 85 | 99 | 4.1^a^ | COMPERA 2.0 3-strata risk  Low risk: 9%  Intermediate risk: 76%  High risk: 16% | COMPERA 2.0 3-strata risk  Low risk: 20%  Intermediate risk: 64%  High risk: 16% | COMPERA 2.0 3-strata risk  Improved: 20%  Unchanged: 69%  Worsened: 11% |  |
|  |  |  |  |  |  |  |  |  |  |  | COMPERA 2.0 4-strata risk  Low risk: 6%  Intermediate-low risk: 24%  Intermediate-high risk: 55%  High risk: 15% | COMPERA 2.0 4-strata risk  Low risk: 17%  Intermediate-low risk: 28%  Intermediate-high risk: 38%  High risk: 17% | COMPERA 2.0 4-strata risk  Improved: 33%  Unchanged: 51%  Worsened: 16% |  |
|  |  |  |  |  |  |  |  |  |  |  | - | Lung transplantation: 1.3% | - |  |
|  |  |  |  |  |  |  |  |  |  |  | - | Prostacyclin analogue use^♠^: 5.0% | - |  |
| Swedish National PH Registry [[12](#_ENREF_12)] | National, systematic, retrospective/ prospective,  7 centres | Sweden | 2002-2015 | Inc/Prev NR  Age NR  *n* = 386 | 60 | 68 | 47 | 75 | 88 | 33-35^b^ | 2015 ESC/ERS risk  - Low risk: 31%  - Intermediate risk: 62%  - High risk: 7% | 2015 ESC/ERS risk  *3-year follow-up*  - Low risk: 39%  - Intermediate risk: 54%  - High risk: 8% *4-year follow-up*  - Low risk: 48%  - Intermediate risk: 45%  - High risk: 8%  *5-year follow-up*  - Low risk: 47%  - Intermediate risk: 49%  - High risk: 4% | - |  |
|  |  |  |  |  |  |  |  |  |  |  | Oxygen use: 17% | Oxygen use  - 3-year FUP: 26%  - 4-year FUP: 22%  - 5-year FUP: 18% | Oxygen use ^†^  - 3-year FUP: +9%  - 4-year FUP: +5%  - 5-year FUP: +1% |  |
|  |  |  |  |  |  |  |  |  |  |  | Prostacyclin analogue use^♠^: 3% | Prostacyclin analogue use^♠^  - 3-year FUP: 1%  - 4-year FUP: 1%  - 5-year FUP: 1% | Prostacyclin analogue use ^†♠^  - 3-year FUP: -2%  - 4-year FUP: -2%  - 5-year FUP: -2% |  |
| Swedish National PH Registry [[13](#_ENREF_13)] | National, systematic, retrospective/ prospective,  7 centres | Sweden | 2008-2016 | Inc (100%)  ≥ 18 yrs  *n* = 530 | - | 65 | - | - | 98 | 4-27^c^ | 2015 ESC/ERS risk  - Low risk: 23%  - Intermediate risk: 67%  - High risk: 10% | 2015 ESC/ERS risk  - Low risk: 29%  - Intermediate risk: 60%  - High risk: 11% | - |  |
|  |  |  |  |  |  |  |  |  |  |  | - | Lung transplantation: 2.5% | - |  |
| Latvian PH Registry [[4](#_ENREF_4)] | National, systematic, prospective, single-centre | Latvia | 2007-2016 | Inc (100%)  ≥ 18 yrs  *n* = 130 | 65 | 73 | 49 | 72 | 99 | 33^d^ | - | Lung transplantation: 0.8% | - |  |
| Swiss PH Registry [[14](#_ENREF_14)] | National, non-systematic, retrospective/ prospective,  13 centres | Switzer-land | 1988-2012 | Inc (95%)  > 18 yrs  *n* = 517 | 57 | 60 | 48 | 75 | 72 | 46^e^ * | - | Lung transplantation: 3.9% | - |  |
| French PAH Registry [[15](#_ENREF_15)] | National, non-systematic,  retrospective/ prospective | France | 2009-2020 | Inc (100%)  ≥ 18 yrs  *n* = 2,879 | 61  ^*†^ | 60 | 45 | 68 | 79 | 5^f^ | COMPERA 2.0 3-strata risk  - Low risk: 16%  - Intermediate: 67%  - High risk: 16% | COMPERA 2.0 3-strata risk  - Low risk: 39%  - Intermediate risk: 53%  - High risk: 8% | COMPERA 2.0 3-strata risk  - Improved: 23%  - Unchanged: 44%  - Worsened: 6%  - Not available: 28% |  |
|  |  |  |  |  |  |  |  |  |  |  | COMPERA 2.0 4-strata  - Low risk: 12%  - Intermediate-low risk: 40%  - Intermediate-high risk: 33%  - High risk: 15% | COMPERA 2.0 4-strata  - Low risk: 33%  - Intermediate-low risk: 38%  - Intermediate-high risk: 23%  - High risk: 6% | COMPERA 2.0 4-strata risk  - Improved: 32%  - Unchanged: 32%  - Worsened: 10%  - Not available: 28% |  |
| Portuguese Registry [[16](#_ENREF_16)] | National, non-systematic, prospective,  5 centres | Portugal | 2008-2010 | Inc (100%)  Adults  *n* = 46 | 43 * | 65 | 51 | 71 | 91^g^ | 12^g^ | Oxygen use: 20% | Oxygen use: 26% | Oxygen use: +6%^†^ |  |
| **North America** | | | | | | | | | | | | | | |
| REVEAL registry [[17](#_ENREF_17)] | National, non-systematic, retrospective/ prospective,  55 centres | US | < 1995-2013 | Inc (26%)  ≥ 19 years  *n* = 2,529 | 53 * | 80 | - | 56 | 59-93^h^ | ≤ 12^h^ | - | - | REVEAL Risk Score  - Worsening: 30%  - No change: 38%  - Improvement: 32% |  |
|  |  |  |  |  |  |  |  |  |  |  | Prostacyclin analogue use^♠^ (%): 15% | Prostacyclin analogue use^♠^ (%)*:* 25% | Prostacyclin analogue use^♠^: +10%^†^ |  |
| REVEAL registry [[18](#_ENREF_18)] | National, non-systematic, retrospective/ prospective,  55 centres | US | < 1995-2013 | Inc (24%)  ≥ 18 years  *n* = 1,426 | - | 80 | - | 51 | - | 36^i^ | BNP (mean): 325 pg/ml | - | BNP  - Increase: 10%  - No change: 83  - Decrease: 7% |  |
|  |  |  |  |  |  |  |  |  |  |  | - | Transplantation^o^: 3.5% | - |  |
| REVEAL registry [[19](#_ENREF_19)] | National, non-systematic, retrospective/ prospective,  55 centres | US | < 1995-2011 | Inc (28%)  ≥ 18 years  *n* = 3,001 | 53 * | 79 | - | 59 | > 90 | ≤ 12^j^ | - | - | Clinical worsening: 45%^j^ |  |
| REVEAL registry [[20](#_ENREF_20)] | National, non-systematic, retrospective/ prospective,  55 centres | US | < 2001-2013 | Inc (100%)  ≥ 18 years  *n* = 710 | 53 | 78 | 50 | 74 | 95 | ≤ 12^k^ | - | - | FC  - Improved: 28%  - Unchanged: 63%  - Worsened: 10% |  |
| PH Association Registry [[21](#_ENREF_21)] | National, non-systematic, retrospective/ prospective,  33 centres | US | < 2015-2018 | Inc (50%)  Adults  *n* = 565 | 56 | 75 | 48 | 59 | 86 | 10^l^ | REVEAL risk  - Low risk: 53%  - Average risk: 16%  - Moderate high risk: 13%  - High risk: 15%  - Very high risk: 3% | REVEAL risk  *Follow-up 1*  - Low risk: 54%  - Average risk: 14%  - Moderate high risk: 11%  - High risk: 12%  - Very high risk: 3%  *Follow-up 2*  - Low risk: 58%  - Average risk: 11%  - Moderate high risk: 12%  - High risk: 12%  - Very high risk: 4%  *Follow-up 3*  - Low risk: 65%  - Average risk: 11%  - Moderate high risk: 6%  - High risk: 11%  - Very high risk: 3%  *Follow-up 4*  - Low risk: 62%  - Average risk: 9%  - Moderate high risk: 9%  - High risk: 9%  - Very high risk: 4% | - |  |
|  |  |  |  |  |  |  |  |  |  |  | WHO FC III/IV: 59% | WHO FC III/IV  *- At follow-up 1:* 46%  *- At follow-up 2:* 40%  *- At follow-up 3:* 32%  *- At follow-up 4:* 48% | WHO FC III/IV ^†^  *- At follow-up 1:* -13%  *- At follow-up 2:* -19%  *- At follow-up 3:* -27%  *- At follow-up 4:* -11% |  |
|  |  |  |  |  |  |  |  |  |  |  | 6MWD (mean): 335m | 6MWD (mean)  *- At follow-up 1:* 361m  *- At follow-up 2:* 373m  *- At follow-up 3:* 377m  *- At follow-up 4:* 347m | 6MWD (mean) ^†^  *- At follow-up 1:* +26m  *- At follow-up 2:* +38m  *- At follow-up 3:* +42m  *- At follow-up 4:* +12m |  |
|  |  |  |  |  |  |  |  |  |  |  | Oxygen use: 38% | Oxygen use  *- At follow-up 1:* 42%  *- At follow-up 2:* 51%  *- At follow-up 3:* 50%  *- At follow-up 4:* 57% | Oxygen use ^†^  *- At follow-up 1:* +4%  *- At follow-up 2:* +13%  *- At follow-up 3:* +12%  *- At follow-up 4:* +19% |  |
|  |  |  |  |  |  |  |  |  |  |  | Prostacyclin analogue use^♠^: 29% | Prostacyclin analogue use^♠^  *- At follow-up 1:* 34%  *- At follow-up 2:* 40%  *- At follow-up 3:* 41%  *- At follow-up 4:* 51% | Prostacyclin analogue use ^†♠^  *- At follow-up 1:* +5%  *- At follow-up 2:* +11%  *- At follow-up 3:* +12%  *- At follow-up 4:* +22% |  |
|  |  |  |  |  |  |  |  |  |  |  | Number of ER visits (median): 1  Number of nights hospitalized (median): 1 | Number of ER visits (median)  - *At follow-up 1: 0*  *- At follow-up 2: 0*  *- At follow-up 3: 0*  *- At follow-up 4: 0*  Number of nights hospitalized (median)  - *At follow-up 1*: *0*  *- At follow-up 2: 0*  *- At follow-up 3: 0*  *- At follow-up 4*: *0* | Number of ER visits (median) ^†^  *- At follow-up 1: -1*  *- At follow-up 2: -1*  *- At follow-up 1: -1*  *- At follow-up 1: -1*  Number of nights hospitalized (median) ^†^  *- At follow-up 1: -1*  *- At follow-up 2: -1*  *- At follow-up 1: -1*  *- At follow-up 1: -1* |  |
|  |  |  |  |  |  |  |  |  |  |  | Emphasis-10 Total Score (mean): 25 | Emphasis-10 Total Score (mean)  *- At follow-up 1: 22*  *- At follow-up 2: 21*  *- At follow-up 3: 20*  *- At follow-up 4: 24* | Emphasis-10 Total Score ^†^ (mean)  *- At follow-up 1: -3*  *- At follow-up 2: -4*  *- At follow-up 3: -5*  *- At follow-up 4: -1* |  |
| **Asia** | | | | | | | | | | | | | | |
| Japan PH Registry [[22](#_ENREF_22), [23](#_ENREF_23)] | National, non-systematic, retrospective,  8 centres | Japan | 2008-2013 | Inc (100%)  ≥ 18 yrs  *n* = 108 | 49  * | 80 | 47 | 65 | 100 | 8^m^ | ESC/ERS risk stratification^m^  - 0 Criteria: 21%  - 1 Criteria: 32%  - 2 Criteria: 32%  - 3 Criteria: 13%  - 4 Criteria: 2% | ESC/ERS risk stratification^m^  - 0 Criteria: 7%  - 1 Criteria: 23%  - 2 Criteria: 33%  - 3 Criteria: 23%  - 4 Criteria: 14% | ESC/ERS risk stratification^m^  - Improved: 59%  - Unchanged: 40%  - Worsened: 10% |  |
|  |  |  |  |  |  |  |  |  |  |  | NYHA FC III/IV: 64% | NYHA FC III/IV: 40% | NYHA FC III/IV: -24% ^†^ |  |
| Japanese Pulmonary Circulation Society Registry [[24](#_ENREF_24)] | Non-national, retrospective/ prospective,  20 centres | Japan | < 2012-2016 | Inc (63%)  Age: -  *n* = 311 | 49* | 77 | 45 | 53 | 92^n^ | 50* | - | Prostacyclin analogue use^♠^: 65%^n^ | - |  |
| 6MWD, 6-min walk distance; BNP, B-type natriuretic peptide; COMPERA, Comparative, Prospective Registry of Newly Initiated Therapies for Pulmonary Hypertension; ER, emergency room; ERS, European Respiratory Society; ESC, European Society of Cardiology; FC, functional class; FUP, follow-up; inc, incident patients; mPAP, mean pulmonary artery pressure; NR or - , not reported; NYHA, New York Heart Association; PH, pulmonary hypertension; PAH, pulmonary arterial hypertension; Prev, prevalent patients; QoL, quality of life; REVEAL, Registry to Evaluate Early and Long-term PAH Disease Management; WHO, World Health Organization; yrs, years.  **Note:** Unless otherwise indicated: age reported as median.  * mean; ^†^ change calculated based on estimates at baseline and follow-up; ^♠^ used as a morbidity outcome as it is potentially associated with disease progression.  ^a^ Baseline defined as at enrolment/before treatment initiation; Follow-up defined as first follow-up from ≥ 12 weeks and up to 12 months after treatment initiation. Proportion of lung transplantation based on overall observation period of 2.6 years; ^b^ Baseline defined as at diagnosis; follow-up at Year 3 (median 33 months); Year 4 (median 33 months); Year 5 (median 35 months); ^c^ Baseline defined as at diagnosis; follow-up for risk assessment is at first follow-up within 12 months (median 4 months); lung transplantation is for overall follow-up (median 27 months); ^d^ Baseline defined as at diagnosis; follow-up at end of follow-up; ^e^ Baseline defined as at enrolment/first visit; follow-up at end of follow-up; ^f^ Baseline defined as at diagnosis; follow-up defined as first follow-up from 3 to 24 months after diagnosis; ^g^ Baseline defined as at study inclusion; follow-up not defined (likely at 1-year follow-up); ^h^ PH Therapy between 59 and 93% for incident and prevalent patients, respectively; Baseline defined as at enrolment, follow-up defined as most recent follow-up within 12 months after enrolment; ^i^ Baseline defined as at enrolment; last follow-up within five years (median three years); ^j^ Baseline defined as at enrolment; follow-up within 12 months after enrolment. Clinical worsening defined as worsening of FC, a ≤ 15% reduction in 6MWD, all-cause hospitalization, or the introduction of a parenteral prostacyclin analogue. Worsening of individual parameters only reported in subgroup analysis for patients who died; ^k^ Baseline defined as at enrolment; follow-up within 12 months after enrolment; ^l^ Baseline not defined (likely enrolment); follow-up at subsequent visits, median follow-up 10 months; ^m^ Median time to first follow-up 293 days. ESC/ERS risk stratification based on Boucly 2017; ^n^ Maximal therapy during follow-up. | | | | | | | | | | | | | | |

## Table S4. Morbidity and QoL for paediatric PH Group 1 patients (4 studies, 4 reports)

| **Study and Reference** | **Classification/ Study design** | **Country** | **Time Period** | **Participants** | **Characteristics at baseline** | | | | **PH Therapy, %** | **Follow-up duration (months)** | **Morbidity and QoL Outcomes** | | | |
| --- | --- | --- | --- | --- | --- | --- | --- | --- | --- | --- | --- | --- | --- | --- |
|  |  |  |  |  | **Age (yrs)** | **Female, %** | **mPAP (mmHg)** | **FC III/IV, %** |  |  | **At baseline** | **At follow-up** | **Change** |  |
| **Europe** | | | | | | | | | | | | | | |
| Dutch National Network for Pediatric PH Registry [[25](#_ENREF_25)] | National, systematic, prospective,  8 centres | Nether-lands | < 2000-2014 | Inc/Prev NR  Children  *n* = 70 | 8 | 66 | 54 | 59 | 100 | ^a^ | - | - | WHO FC  - Worsening: 63% ^a^ |  |
|  |  |  |  |  |  |  |  |  |  |  | - | - | WHO FC or 6MWD  - Worsening: 71% |  |
|  |  |  |  |  |  |  |  |  |  |  | - | - | Clinical worsening composite: 84% ^a^ |  |
|  |  |  |  |  |  |  |  |  |  |  | - | Lung-transplantation: 10% | - |  |
|  |  |  |  |  |  |  |  |  |  |  | Prostacyclin analogue use^♠^: 19% | Prostacyclin analogue use^♠^: 37% | Prostacyclin analogue use^♠^: +18% ^†^ |  |
|  |  |  |  |  |  |  |  |  |  |  | - | Hospitalization: 54% | - |  |
| French pediatric PAH Registry [[26](#_ENREF_26)] | National, non-systematic, retrospective/ prospective,  21 centres | France | < 2005-2008 | Inc (30%)  ≥ 28d to  ≤ 18yrs  *n* = 50  RHC^e^ (86%) | 9* | 48 | 59 | 28 | 82 | 23^b^ |  |  | WHO FC  - Improved: 25%  - Unchanged: 48%  - Worsened: 27% |  |
|  |  |  |  |  |  |  |  |  |  |  | 6MWD (mean): 421m | 6MWD (mean): 448m | 6MWD (mean): +27m ^†^ |  |
|  |  |  |  |  |  |  |  |  |  |  | Prostacyclin analogue use^♠^: 28% | Prostacyclin analogue use^♠^: 24% | Prostacyclin analogue use^♠^: -4% ^†^ |  |
|  |  |  |  |  |  |  |  |  |  |  | CHQ-PF50 score (mean)  GH: 52  PF: 59  REB: 63  RP: 56  BP: 69  BE: 75  MH: 60  SE: 69  PE: 55  PT: 65  FA: 77  FaC: 74 | CHQ-PF50 score (mean)  GH: 61  PF: 62  REB: 77  RP: 70  BP: 83*  BE: 77  MH: 70*  SE: 64  PE: 60  PT: 64  FA: 80  FaC: 77  **p < 0.05 at follow-up* | CHQ-PF50 score (mean) ^†^  GH: +9  PF: +3  REB: +14  RP: +14  BP: +14  BE: +2  MH: +10  SE: -5  PE: +5  PT: -1  FA: +3  FaC: +3 |  |
| Polish Registry of PH [[27](#_ENREF_27)] | National, systematic, retrospective/ prospective,  8 centres | Poland | < 2018-2019 | Inc (13%)  ≥ 3m to ≤ 18yrs  *n* = 80 | 10* | 50 | 48 | 31 | 98 | 17^c^ | Prostacyclin analogue use^♠^: 4% | Prostacyclin analogue use^♠^: 13% | Prostacyclin analogue use^♠^: +9% ^†^ |  |
|  |  |  |  |  |  |  |  |  |  |  | - | Hospitalization: 11% | - |  |
| **North America** | | | | | | | | | | | | | | |
| REVEAL registry - pediatric arm [[28](#_ENREF_28)] | National, non-systematic, retrospective/ prospective,  26 centres | US | < 1995-2010 | Inc (14%)  ≥ 3m to ≤ 18yrs  *n* = 216 | 15 | 64 | 56^#^ | 28 | 85 | 42^d^ | - | Transplantations: 3% | - |  |
| 6MWD, 6-min walk distance; BE, general behavior; BP, bodily pain/discomfort; CHQ-P50, Patient-Reported 50-Item Child Health Questionnaire; FA, family limitations in activities; FC, functional class; FaC, family cohesion; GH, general health; inc, incident patients; m, months; MH, mental health; PAH, pulmonary arterial hypertension; PH, pulmonary hypertension; mPAP, mean pulmonary artery pressure; NR or - , not reported; PE, parental impact-emotional; PF, physical functioning; Prev, prevalent patients; PT, parental impact-time; QoL, quality of life; yrs, years ; REB, role/social-emotional-behavioral; REVEAL, Registry to Evaluate Early and Long-term PAH Disease Management; RHC, right-heart catheterization; RP, role/social-physical; SE, self-esteem; WHO, World Health Organization.  **Notes:** Unless otherwise indicated: age reported as median  * mean; ^†^ change calculated based on estimates at baseline and follow-up; ^#^ reported at diagnosis; ^♠^ used as a morbidity outcome as it is potentially associated with disease progression.  ^a^ Baseline defined as at treatment initiation/enrolment. Follow-up defined as at last visit. Total follow-up 276 person-years. For worsening of FC or 6MWD, FC IV was always regarded as a functional deterioration event. Clinical worsening composite endpoint defined as: death, lung transplantation, hospitalization, initiation of IV prostacyclin analogues, functional deterioration, worsening WHO FC or decreased 6MWD (> = 15%); ^b^ Baseline defined as at enrolment. Follow-up assessments conducted at six months, one year and two years. Follow-up defined as at latest available assessment; ^c^ Baseline defined as at enrolment. Follow-up defined as during follow-up; ^d^ Baseline defined as at enrolment. Follow-up defined as during follow-up. | | | | | | | | | | | | | | |

## Supplementary Method 1. Search strategy

We conducted an electronic database search on 25 Nov 2021, comprised of three parts. Firstly, we carried out a systematic search of articles and conference abstracts via OvidSP (Medline^®^ and Embase^®^), based on the PICO (Population, Intervention, Comparator and Outcome) approach [[1](#_ENREF_1)]. We used three key search blocks, including: 1) population (patients with PH, including PAH); 2) outcomes (survival, morbidity, and/or QoL), and; 3) study design (observational studies, including registries, cohorts, databases, and chart reviews). Search terms and the number of hits returned for each component are summarized below. We did not restrict the search by year of publication or language, to improve search sensitivity. We employed a semi-automated procedure for deduplication.

Secondly, we searched clinicaltrials.gov and encepp.eu for relevant database entries using the keyword ‘pulmonary hypertension’ and restricted the results to observational studies only. We also conducted a grey literature search via Google, to identify unpublished reports (e.g., annual registry reports). Websites of PH registries and key patient PH organizations were also searched. Finally, we searched bibliographies of seminal reviews of PH registries [[2-5](#_ENREF_2)] and of studies included in the review. We exported all records identified by the three searches to a Microsoft Excel spreadsheet for screening.

**Search strategy (search date 25 Nov 2021) utilized for electronic search including via EMBASE^®^ and Ovid MEDLINE^®^.**

| # | Terms | Records |
| --- | --- | --- |
| 1 | pulmonary hypertension/; or (pulmonary hypertension or pulmonary arterial hypertension).ti.ab. | 156,288 |
| 2 | (survival or mortality).ti.ab. | 4,277,807 |
| 3 | (6MWD or 6-min* walk distance or 6 min* walk distance or 6MWT or 6-min* walk test or 6 min* walk test or WHO FC or WHO functional class or NYHA FC or NYHA functional class or prostanoid or prostacyclin or oxygen therapy or treatment escalation or lung transplant* or risk assessment or risk stratification or risk score? Or risk level? Or clinical worsening or hospitalization).ti.ab. | 891,887 |
| 4 | (quality of life or QoL or patient reported outcome? Or patient-reported outcome? Or PRO).ti.ab. | 1,398,596 |
| 5 | (observational or registry or cohort or database or hospital records or chart review).ti.ab. | 3,279,841 |
| 6 | 2 or 3 or 4 | 6,093,807 |
| 7 | 1 and 5 and 6 | 9,412 |
|  | Manual duplicate removal with Endnote 20 *(using fields author, year, title, journal and pages in multiple iterations)* | 6,872 |
| 6MWD**,** 6-min walk distance; 6MWT, 6-min walk test; FC, functional class; NYHA, New York Heart Association; PRO, patient reported outcome; QoL, quality of life; ti.ab, terms in the title or abstract fields; WHO, World Health Organization. | | |

## Supplementary Method 2. Selection of studies

We defined eligibility criteria in line with the aims of this review, and to ensure that the outcome estimates were based on a well-defined, comparable, and representative PAH population. The selection of studies for inclusion into the review was undertaken by a single reviewer (SR). The titles and abstracts of articles, as well as the names and descriptions of database entries or other reports were screened to identify potentially relevant studies, based on the eligibility criteria presented in **Table 1**.

The population of interest was defined as patients of any age with PAH according to the 3^rd^ WSPH Venice classification or later [[6](#_ENREF_6)], and diagnosed by RHC, as recommended by ESC/ERS guidelines [[7](#_ENREF_7)]. Studies with mixed diagnosis by RHC and echocardiography were also included. We defined survival as the primary outcome of interest; secondary outcomes included a wider range of indicators grouped under morbidity (including risk scores, hospitalization, FC, 6MWD, BNP/NT-proBNP, transplantation, oxygen use, and prostacyclin analog use potentially associated with disease progression), as well as QoL or patient-reported outcomes (PRO) measures. Only observational studies where population-based representativeness could reasonably be assumed were considered relevant, including multi-center or single-center studies with nationwide catchment areas.

We excluded studies not covering the population, outcomes or study type of interest; **see** **Table 1**. PAH studies were excluded if they covered ≤ 3 PAH aetiologies, as they could not reasonably be considered to be representative of the overall PAH population. We also excluded studies covering only selected subpopulations (e.g., pregnant women, transplantation candidates).

We then conducted a full text review of potentially relevant studies to confirm final eligibility. Number of centers and geographic catchment areas were identified, single-center studies with no explicitly stated national representativeness were excluded. Reports on the same study were identified and linked. To avoid duplicate data reporting, reports covering the same study population and reporting only outcomes for the same or earlier time period as well as reports on patients already covered by a larger study included in the review were excluded.

## Supplementary Method 3. Data collection

Data were collected across four domains: 1) general information; 2) study population; 3) baseline characteristics, and; 4) outcomes at follow-up. If no indication of age group was reported by authors of a study, age group was classified as adult/all ages. We collected baseline characteristics at diagnosis or enrollment, as reported by authors. 1-, 3-, and 5-year survival estimates were collected as reported by authors or extracted from Kaplan-Meier curves using Engauge Digitizer 12.0 [[8](#_ENREF_8)]. For morbidity and QoL outcomes, data collected included estimates at baseline and follow-up or change from baseline to follow-up. The variables outlined in the table below were collected and stored in a standardized data collection form to ensure consistent data extraction across studies. We developed data collection forms within Microsoft Excel and included pre-defined data fields covering the four domains. Data extraction was performed by SR.

| **Domain/ Variable** | **Description** |
| --- | --- |
| **(1) General Information** | |
| Author/Year | - Name of first author and year of publication. |
| Name/Title | - Name and title of the study. If not available, name of institution where the study was conducted. |
| Acronym | - Acronym of the study, if available. |
| Type of Study | - Three study types were differentiated based on their design and as described by the authors: *(1) Registries; (2) Cohorts; (3) Chart Reviews.* |
| Data collection | - Methods for data collection employed in study, *prospective* for entirely prospective data collection, *retrospective* for entirely retrospective data collection and *prospective/retrospective* if both methods are employed. |
| Classification | - Classification of studies regarding their population-level representativeness, considering geographical coverage and completeness of coverage of centres. Categories include:  *(1) National, systematic (2) National, non-systematic (3) Non-national (4) Multi-national.* |
| Centres | - Number of centres participating in study, defined as either referral centres or medical centres/hospitals where patients receive diagnosis and treatment. |
| Total Size | - Most recent total number of PH patients included in study, from key reference, substudies or supporting references. |
| Country | - Country or countries where the study was conducted. |
| Region | - Region of the world, including *(1) Africa, (2) Europe, (3) Latin America, (4) North America, (5) Asia, (6) Pacific, (7) International, (8) ROW.* |
| Description | - Top-line description of the study/data source. |
| Study period | - Range, in calendar years, from enrolment of first participant through enrolment of last participant or date of latest follow-up, whichever is later and available. Diagnosis dates may be taken as a proxy for enrolment dates if enrolment dates are not reported. |
| Aim of Substudy | - If multiple reports/analysis for the same data source are reviewed, aims of each substudy are described separately. |
| **(2) Population** | |
| PH Group | - Group of PH (WHO Group 1, 2, 3, 4, 5) covered in study. |
| PH Diagnosis Criteria | - Type of diagnostic method and the WSPH classification system used for diagnosis of patients. As per selection criteria, the diagnostic method must be RHC using the 3^rd^ WSPH Venice classification system or later:   *(1) 3^rd^ WSPH Venice  (2) 4^th^ WSPH Dana Point (3) 5^th^ WSPH Nice  (4) 6^th^ WSPH Nice.* |
| Incident/Prevalent | - Specifies whether patients included in the study are: (1) *Incident cases*, i.e. Newly diagnosed patients (2) *Prevalent cases*, i.e. Previously diagnosed patients  *(3) Incident and prevalent cases*, i.e. Both type of cases. |
| Time period | - Range, in calendar years, from earliest diagnosis date through latest diagnosis date or date of latest follow-up, whichever is later and available. Diagnosis rather than enrolment date was chosen as describing the most accurate reference period regarding treatment availability, for interpretation of patient characteristics/outcomes, and to allow uniform description across different types of studies (prospective, retrospective/prospective, retrospective) - For studies that do not describe earliest diagnosis date, the start of prospective enrolment and/or retrospective data collection will be supplemented with the prefix “<” to indicate that patients with earlier diagnosis dates are included but earliest diagnosis date is unknown. In most prospective studies with incident patients, however, enrolment date largely corresponds to diagnosis date - If date of final follow-up is after date of latest diagnosis but not described, this is indicated with the postfix “+” indicating that patients were follow-up beyond the latest diagnosis date. |
| Age group | - Age group of patients included in study in years. If no years reported, an approximation of the age group is reported (i.e. adults, children, all age groups). If no indication of age group is given by authors, age group is not reported (i.e. “NR”) and for the report assumed as adults. |
| Inclusion Criteria | - Explicit inclusion criteria of the study population as stated by authors, in addition to RHC criteria. |
| Exclusion Criteria | - Explicit exclusion criteria of the study population as stated by authors. |
| Sample Size | - Sample size of the study population covered in analysis. |
| Sample Distribution | - Description of the population included in the study at baseline. |
| **(3) Baseline characteristics** | |
| Demographics | - Summary statistics of age and gender, including age at onset of symptoms, diagnosis and enrolment, if available. |
| Functional Class | - New York Heart Association (NYHA)/World Health Organization (WHO) Functional Class (FC) at baseline. Estimates are reported as proportions for each class, as reported for baseline characteristics of sample. |
| Haemodynamics | - Description of haemodynamic status at baseline using signal indicator mPAP. |
| 6MWD | - 6 min-walk distance in meters at baseline. |
| Morbidity | - Other morbidity outcomes, such as oxygen therapy, prostacyclin use potentially associated with disease progression, or lab variables at baseline. |
| Risk assessment | - Type of risk assessment tool used. Estimates are risk groups or stratification of patients at baseline. If risk assessment is only used for stratification of other outcomes this is indicated. A top-line description of risk assessment tools, including variables covered, scoring algorithm, scores and interpretation is available in the sheet “Risk Assessment & QoL”. |
| Hospitalization | - Prevalence of previous hospitalization at baseline. |
| Quality of life | - QoL tools used. Estimates are for scores as measured at baseline. |
| **(4) Outcomes at Follow-up** | |
| Follow-up duration | - Median or mean follow-up duration of patients. |
| Survival/ Mortality | - Survival or mortality estimates are provided for each year as reported in the reference. If not reported in text form and Kaplan-Meier curves are presented, estimates are directly extracted from curves using Engauge Digitizer (indicated with “ * “). Methods for survival calculation are specified in brackets, including type of events counted (deaths or other events such as transplantation or clinical worsening) and timepoints used (e.g. From enrolment or diagnosis). If not described otherwise, it is assumed that survival calculation is based on all-cause mortality. |
| Morbidity | - Morbidity outcomes, including FC, 6MWD, NT-proBNP/BNP, oxygen use, prostacyclin analogue use potentially associated with disease progression, clinical worsening at follow-up or change between baseline and follow-up. Prevalence of transplantation at follow-up. |
| Risk assessment | - Type of risk assessment tool used. Estimates are risk groups or strata at follow-up, or changes between baseline and follow-up. A brief description of risk assessment tools, including variables covered, scoring algorithm, scores and interpretation is available in the sheet *“Risk Scores”.* |
| Hospitalization | - Prevalence of hospitalization during follow-up, time to hospitalization, or hospitalization rate at follow-up. |
| Quality of life | - QoL instruments used. Estimates are for scores as measured at follow-up or change in scores from baseline to follow-up. |
| 6MWD, 6-min walk distance; BNP, B-type natriuretic peptide; FC, functional class; mPAP, mean pulmonary artery pressure; NR, not reported; NT-proBNP, N-terminal pro-brain natriuretic peptide; NYHA, New York Heart Association; PH, pulmonary hypertension; QoL, quality of life; RHC, right-heart catheterization; ROW, rest of world; WHO, World Health Organization; WSPH, World Symposium of Pulmonary Hypertension. | |

## Supplementary Method 4. Assessment of representativeness

We assessed representativeness of studies using an adapted classification system based on definitions developed by Leber et al. (2021) [[3](#_ENREF_3)]. and outlined in table below. Studies that covered all national PH expert or referral centers were classified as ‘national, systematic’. Studies that had large geographic coverage of most regions nationally and/or were described by the authors as ‘national’, but with incomplete coverage of all centers on a national level, were classified as ‘national, non-systematic’. Other studies were classified as ‘non-national’; – in the case of studies covering multiple countries, they were classified ‘multi-national’. Whilst Leber et al. (2021) [[3](#_ENREF_3)] also considered mandatory (as opposed to voluntary) participation of centers within a registry as criteria for systematic studies, we did not consider this as it was not reported by most authors.

| *Classification* | *Geographical Coverage* | *Coverage of Centres* | *Participant registration* |
| --- | --- | --- | --- |
| National, systematic | Geographical catchment area defined on a national level: (1) majority of regions/states are covered; and/or (2) study described as “national” by authors. | All national PH expert or referral centres (but not necessarily all hospitals) covered, multi-centre or single-centre with national coverage*. | Voluntary enrolment with patient consent or mandatory enrolment without consent (usually in retrospective studies where consent is waived)^¶^. |
| National, non-systematic |  | Some but not all national PH expert or referral centres covered; multi-centre. |  |
| Non-national | No geographical catchment area defined or not described as national. | Multiple PH expert or referral centres^†^ but not all centres are covered. |  |
| Multi-national | Multiple countries participating with no geographical catchment area within each participating country defined. | Multiple PH expert or referral centres^†^ of multiple countries covered; no complete coverage of centres for countries. |  |
| PH, pulmonary hypertension.  *For classification of systematic coverage, Leber (2021)[[3](#_ENREF_3)] also assess voluntary or mandatory participation of centres as well as type of healthcare system and referral pathways. However, since mostly not reported by authors, this was not considered in our representativeness assessment. ^†^Due to multiple centres participating, some population-level representativeness is inferred. ^¶^Only used in representativeness assessment but not for classification/categorization. | | | |

## Supplementary Method 5. Data reporting

We characterized all included studies with respect to age, gender, functional class, mPAP, PH therapy, representativeness, country, time period (patients’ earliest diagnosis to the latest follow-up date) and number of participants, proportion of incident, and prevalent patients. We considered these to be key factors that may reveal patterns in outcomes data. We reported survival in a standardized form as 1- 3-, and 5-year probabilities to survive. For morbidity and/or QoL outcomes, we reported relevant estimates at baseline, follow-up, and change from baseline to follow-up. If not reported, we calculated change as the absolute difference between estimates at baseline and follow-up. In case multiple follow-up points were available, we always calculated change from baseline. In case the same type of outcome was reported by multiple records from the same study, we only reported outcomes for the most recent time period and/or for incident patients. Whenever available, we reported characteristics and outcomes for incident patients to minimize survival bias.

## Supplementary Method 6. Data synthesis

We conducted a narrative synthesis of the results separately by type of outcome (survival and morbidity/QoL outcomes) and by age group (adult and pediatric patients). We further grouped studies by geographic region (Africa, Asia, Europe, Latin America, North America, Pacific, International) due to geographic variation in healthcare systems and availability of treatments.

We conducted post-hoc subgroup meta-analyses to explore differences in adult survival by relevant subgroups that might explain heterogeneity, including geographic region, disease severity, study period, and study representativeness, whenever there were three or more studies in each subgroup. We operationalized study period as the mid-year of diagnosis or enrollment into the study (ie. 2010 if earliest and latest date of diagnosis was 2005 and 2015) and used equal-sized subgroups for analyses (ie. 15 studies with adult 1-year survival were grouped into Years 2005-2011 with 8 studies and Years 2012-2017 with 7 studies). Geographic region and representativeness (systematic compared to non-systematic or non-national coverage) was defined as previously described. We used FC at baseline (ie. proportion of patients with FC III/IV) as a proxy for disease severity with equal-sized subgroups. Subgroup meta-analyses were conducted using random effects models with inverse Freeman-Tukey transformed proportions and the restricted maximum likelihood estimator for between-study variance [[9](#_ENREF_9), [10](#_ENREF_10)]. Analyses were conducted using Stata 18.0 (Stata Corp, College Station, Texas).

**References**

1. Higgins JTJ, M. CJC, Li T, Page M, Welch V: **Cochrane Handbook for Systematic Reviews of Interventions version 6.3** 2022.

2. Emmons-Bell S, Johnson C, Boon-Dooley A, Corris PA, Leary PJ, Rich S, Yacoub M, Roth GA: **Prevalence, incidence, and survival of pulmonary arterial hypertension: A systematic review for the global burden of disease 2020 study.** *Pulm Circ* 2022, **12:**e12020.

3. Leber L, Beaudet A, Muller A: **Epidemiology of pulmonary arterial hypertension and chronic thromboembolic pulmonary hypertension: identification of the most accurate estimates from a systematic literature review.** *Pulm Circ* 2021, **11:**2045894020977300.

4. Skride A, Sablinskis K, Lejnieks A, Rudzitis A, Lang I: **Characteristics and survival data from Latvian pulmonary hypertension registry: comparison of prospective pulmonary hypertension registries in Europe.** *Pulm Circ* 2018, **8:**2045894018780521.

5. Swinnen K, Quarck R, Godinas L, Belge C, Delcroix M: **Learning from registries in pulmonary arterial hypertension: pitfalls and recommendations.** *Eur Respir Rev* 2019, **28:**190050.

6. Galiè N, Torbicki A, Barst R, Dartevelle P, Haworth S, Higenbottam T, Olschewski H, Peacock A, Pietra G, Rubin LJ, et al: **Guidelines on diagnosis and treatment of pulmonary arterial hypertension. The Task Force on Diagnosis and Treatment of Pulmonary Arterial Hypertension of the European Society of Cardiology.** *Eur Heart J* 2004, **25:**2243-2278.

7. Humbert M, Kovacs G, Hoeper MM, Badagliacca R, Berger RMF, Brida M, Carlsen J, Coats AJS, Escribano-Subias P, Ferrari P, et al: **2022 ESC/ERS Guidelines for the diagnosis and treatment of pulmonary hypertension.** *Eur Heart J* 2022, **43:**3618-3731.

8. **Engauge Digitizer Software** [<http://markummitchell.github.io/engauge-digitizer>]

9. Hedges LV: **A random effects model for effect sizes.** *Psychological Bulletin* 1983, **93:**388-395.

10. Miller JJ: **The Inverse of the Freeman – Tukey Double Arcsine Transformation.** *The American Statistician* 1978, **32:**138-138.

11. Hoeper MM, Pausch C, Olsson KM, Huscher D, Pittrow D, Grunig E, Staehler G, Vizza CD, Gall H, Distler O, et al: **COMPERA 2.0: A refined 4-strata risk assessment model for pulmonary arterial hypertension.** *Eur Respir J* 2021.

12. Kylhammar D, Hjalmarsson C, Hesselstrand R, Jansson K, Kavianipour M, Kjellström B, Nisell M, Söderberg S, Rådegran G: **Predicting mortality during long-term follow-up in pulmonary arterial hypertension.** *ERJ Open Res* 2021, **7**.

13. Kylhammar D, Kjellström B, Hjalmarsson C, Jansson K, Nisell M, Söderberg S, Wikström G, Rådegran G: **A comprehensive risk stratification at early follow-up determines prognosis in pulmonary arterial hypertension.** *Eur Heart J* 2018, **39:**4175-4181.

14. Mueller-Mottet S, Stricker H, Domenighetti G, Azzola A, Geiser T, Schwerzmann M, Weilenmann D, Schoch O, Fellrath JM, Rochat T, et al: **Long-term data from the Swiss pulmonary hypertension registry.** *Respiration* 2015, **89:**127-140.

15. Boucly A, Weatherald J, Savale L, de Groote P, Cottin V, Prevot G, Chaouat A, Picard F, Horeau-Langlard D, Bourdin A, et al: **External validation of a refined 4-strata risk assessment score from the French pulmonary hypertension Registry.** *Eur Respir J* 2021.

16. Baptista R, Meireles J, Agapito A, Castro G, da Silva AM, Shiang T, Gonçalves F, Robalo-Martins S, Nunes-Diogo A, Reis A: **Pulmonary hypertension in Portugal: first data from a nationwide registry.** *Biomed Res Int* 2013, **2013:**489574.

17. Benza RL, Miller DP, Foreman AJ, Frost AE, Badesch DB, Benton WW, McGoon MD: **Prognostic implications of serial risk score assessments in patients with pulmonary arterial hypertension: a Registry to Evaluate Early and Long-Term Pulmonary Arterial Hypertension Disease Management (REVEAL) analysis.** *J Heart Lung Transplant* 2015, **34:**356-361.

18. Frantz RP, Farber HW, Badesch DB, Elliott CG, Frost AE, McGoon MD, Zhao C, Mink DR, Selej M, Benza RL: **Baseline and Serial Brain Natriuretic Peptide Level Predicts 5-Year Overall Survival in Patients With Pulmonary Arterial Hypertension: Data From the REVEAL Registry.** *Chest* 2018, **154:**126-135.

19. Frost AE, Badesch DB, Miller DP, Benza RL, Meltzer LA, McGoon MD: **Evaluation of the predictive value of a clinical worsening definition using 2-year outcomes in patients with pulmonary arterial hypertension: a REVEAL Registry analysis.** *Chest* 2013, **144:**1521-1529.

20. Farber HW, Miller DP, Poms AD, Badesch DB, Frost AE, Muros-Le Rouzic E, Romero AJ, Benton WW, Elliott CG, McGoon MD, Benza RL: **Five-Year outcomes of patients enrolled in the REVEAL Registry.** *Chest* 2015, **148:**1043-1054.

21. Borgese M, Badesch D, Bull T, Chakinala M, DeMarco T, Feldman J, Ford HJ, Grinnan D, Klinger JR, Bolivar L, et al: **EmPHasis-10 as a measure of health-related quality of life in pulmonary arterial hypertension: data from PHAR.** *Eur Respir J* 2021, **57**.

22. Tamura Y, Kumamaru H, Satoh T, Miyata H, Ogawa A, Tanabe N, Hatano M, Yao A, Abe K, Tsujino I, et al: **Effectiveness and Outcome of Pulmonary Arterial Hypertension-Specific Therapy in Japanese Patients With Pulmonary Arterial Hypertension.** *Circ J* 2017, **82:**275-282.

23. Tamura Y, Kumamaru H, Abe K, Satoh T, Miyata H, Ogawa A, Tanabe N, Hatano M, Yao A, Tsujino I, et al: **Improvements in French risk stratification score were correlated with reductions in mean pulmonary artery pressure in pulmonary arterial hypertension: a subanalysis of the Japan Pulmonary Hypertension Registry (JAPHR).** *BMC Pulm Med* 2021, **21:**28.

24. Kozu K, Sugimura K, Ito M, Hirata KI, Node K, Miyamoto T, Ueno S, Watanabe H, Shimokawa H, Japanese Pulmonary Circulation Study G: **Current status of long-term prognosis among all subtypes of pulmonary hypertension in Japan.** *Int J Cardiol* 2020, **300:**228-235.

25. Ploegstra MJ, Arjaans S, Zijlstra WMH, Douwes JM, Vissia-Kazemier TR, Roofthooft MTR, Hillege HL, Berger RMF: **Clinical Worsening as Composite Study End Point in Pediatric Pulmonary Arterial Hypertension.** *Chest* 2015, **148:**655-666.

26. Fraisse A, Jais X, Schleich JM, di Filippo S, Maragnès P, Beghetti M, Gressin V, Voisin M, Dauphin C, Clerson P, et al: **Characteristics and prospective 2-year follow-up of children with pulmonary arterial hypertension in France.** *Arch Cardiovasc Dis* 2010, **103:**66-74.

27. Kwiatkowska J, Zuk M, Migdal A, Kusa J, Skiba E, Zygielo K, Przetocka K, Werynski P, Banaszak P, Rzeznik-Bieniaszewska A, et al: **Children and Adolescents with Pulmonary Arterial Hypertension: Baseline and Follow-Up Data from the Polish Registry of Pulmonary Hypertension (BNP-PL).** *J Clin Med* 2020, **9**.

28. Barst RJ, McGoon MD, Elliott CG, Foreman AJ, Miller DP, Ivy DD: **Survival in childhood pulmonary arterial hypertension: insights from the registry to evaluate early and long-term pulmonary arterial hypertension disease management.** *Circulation* 2012, **125:**113-122.
